# A critical residue in a conserved RBD epitope determines neutralization breadth of pan-sarbecovirus antibodies with recurring YYDRxxG motifs

**DOI:** 10.1128/mbio.00606-25

**Published:** 2025-07-31

**Authors:** Saskia C. Stein, George Ssebyatika, Tim Benecke, Luisa Ströh, Manoj K. Rajak, Benjamin Vollmer, Sarah Menz, Ja-Yun Waldmann, Sarah N. Tipp, Okechukwu Ochulor, Elisabeth Herold, Britta Schwarzloh, Doris Mutschall, Jasmin Zischke, Talia Schneider, Imke Hinrichs, Rainer Blasczyk, Hannah Kleine-Weber, Markus Hoffmann, Florian Klein, Franziska K. Kaiser, Mariana Gonzalez-Hernandez, Federico Armando, Malgorzata Ciurkiewicz, Georg Beythien, Stefan Pöhlmann, Wolfgang Baumgärtner, Kay Gruenewald, Albert Osterhaus, Thomas F. Schulz, Thomas Krey, Guido Hansen

**Affiliations:** 1 Institute of Virology, Hannover Medical School 686461, Hannover, Germany; 2 Institute of Biochemistry, Center of Structural and Cell Biology in Medicine, University of Lübeck 240404, Lübeck, Germany; 3 Centre for Structural Systems Biology, Hamburg, Germany; 4 Department of Chemistry, University of Hamburg 200982, Hamburg, Germany; 5 Leibniz Institute of Virology 28367, Hamburg, Germany; 6 German Center for Infection Research, Partner Site Hannover-Braunschweig, Hannover, Germany; 7 Institute of Transfusion Medicine and Transplant Engineering, Hannover Medical School, Hannover, Germany; 8 Faculty of Biology and Psychology, German Primate Center, Leibniz Institute for Primate Research, University of Göttingen 98900, Göttingen, Germany; 9 Laboratory of Experimental Immunology, Institute of Virology, University of Cologne 686814, Cologne, Germany; 10 German Center for Infection Research, Partner Site Bonn-Cologne, Cologne, Germany; 11 Center for Molecular Medicine Cologne (CMMC), University of Cologne 553477, Cologne, Germany; 12 Research Center for Emerging Infections and Zoonoses, University of Veterinary Medicine Hannover, Foundation, Hannover, Germany; 13 Department of Pathology, University of Veterinary Medicine Hannover, Foundation 26556 , Hannover, Germany; 14 Center for Systems Neuroscience, University of Veterinary Medicine Hannover, Foundation, Hannover, Germany; 15 Excellence Cluster 2155 RESIST, Hannover Medical School, Hannover, Germany; 16 Global Virus Network, Center of Excellence, University of Veterinary Medicine, Hannover, Germany; 17 German Center for Infection Research, Partner Site Hamburg-Lübeck-Borstel-Riems, Braunschweig, Germany; Charité-Universitätsmedizin Berlin, Berlin, Germany

**Keywords:** neutralizing antibody, neutralization escape, receptor-binding domain, sarbecovirus, SARS-CoV-2

## Abstract

The emergence of pandemic coronaviruses remains a global health concern, highlighting the need for broadly neutralizing antibodies (bnAbs) that can target multiple sarbecoviruses. In this study, we isolated and characterized a novel antibody, pT1679, that demonstrates exceptional neutralization breadth. The antibody prevented infection with SARS-CoV-2 variants of concern, such as Omicron BA.1, and effectively neutralized pseudotyped viruses displaying S proteins from many SARS-CoV-2 variants and various bat and pangolin sarbecoviruses, including both SARS-CoV-like and SARS-CoV-2-like viruses. In addition, pT1679 reduced the viral load in the lung of infected Syrian hamsters and prevented the severe lung pathology typical for SARS-CoV-2 infections. The cryo-electron microscopy structure of pT1679 in complex with SARS-CoV-2 S revealed that the antibody employs a YYDRxxG motif to recognize a highly conserved epitope on the RBD. Through detailed structural analysis, mutagenesis studies, and binding assays, we identified RBD residue 384 as a critical determinant of antibody recognition. Structure-function analyses of several related bnAbs, such as COVA1-16, allowed for the classification of YYDRxxG antibodies into two distinct groups that differ in neutralization breadth. Our findings provide crucial insights into the molecular basis of broad *Sarbecovirus* neutralization and offer strategic guidance for selecting therapeutic antibodies in preparation for future *Sarbecovirus* outbreaks.

**IMPORTANCE:**

The threat of emerging coronaviruses demands therapeutic strategies capable of targeting both current and future circulating viruses. We report the discovery and characterization of pT1679, a broadly neutralizing antibody that demonstrates cross-reactivity against diverse sarbecoviruses, including SARS-CoV, SARS-CoV-2 variants, and related viruses from bats and pangolins. pT1679 targets a highly conserved epitope via a YYDRxxG motif in the paratope, with RBD residue 384 serving as a critical determinant of recognition. Our analysis allows for a classification of YYDRxxG antibodies, providing a framework for predicting antibody effectiveness against emerging sarbecoviruses.

## INTRODUCTION

In view of the emergence of three highly pathogenic human betacoronaviruses with severe epidemic and pandemic potential over the last two decades and the sporadic transmission of additional animal coronaviruses to humans (1, 2), the likelihood of another zoonotic outbreak of an as yet unknown coronavirus is high. Identifying and stocking human bnAbs capable of inhibiting a variety of related betacoronaviruses could therefore represent an important contribution towards “epidemic and pandemic preparedness.”

Many previous reports have helped to elucidate the structural requirements for antibody-mediated SARS-CoV-2 neutralization. Each protomer within the SARS-CoV-2 S protein trimer comprises an S1 subunit, which facilitates binding to the cellular receptor ACE2 via a receptor-binding domain (RBD), and an S2 subunit, which drives fusion of the viral envelope with a target cell membrane and harbors a fusion peptide and transmembrane domain. The RBDs are mobile and may bind ACE2 only when in an “up” conformation compared to the “down” RBD conformation of the prefusion S trimer. Neutralization epitopes on the SARS-CoV-2 S RBD have been classified on the basis of structural and functional data (3–6). Barnes class 1 antibodies all have a similar angle of approach to bind to a group of epitopes heavily overlapping with the ACE2 binding site. Class 2 antibodies also recognize adjacent epitopes that overlap with the ACE2 binding site but are more heterogeneous in terms of germline usage and angle of approach. The epitopes of class 3 antibodies do not overlap with the ACE2 binding site and usually include the N-glycosylation site at position 343. Class 4 antibodies bind to epitopes at the inner face of the RBD, without significant overlap with the ACE2 binding site, likely competing with ACE2 by steric hindrance instead. A few reported antibodies do not fall into any of these four classes, and their binding mode to the RBD has also been structurally characterized (7, 8). Among these, a few antibodies have been reported to target the more conserved β-strand core of the RBD with an extended CDR2 region and neutralize SARS-CoV-2 by destroying the S trimer without interfering significantly with its binding to ACE2 (9–15). Most class 1 and 2 antibodies tend to have a narrow neutralization range, and SARS-CoV-2 variants resistant to neutralization with class 1 and 2 antibodies emerged rapidly (16), although recently a class 1 antibody with exceptional breadth has been reported (17). In contrast, some class 3 antibodies and antibodies targeting the conserved β-strand core of the RBD retained the ability to neutralize emerging SARS-CoV-2 Omicron variants (16). While numerous human antibodies effectively neutralize SARS-CoV-2 and its variants, cross-neutralization against related sarbecoviruses—predominantly found in diverse bat species—remains limited (18). Recently, a subset of class 4 antibodies has been characterized that share unique molecular properties (19–28). These antibodies feature an elongated CDRH3 with a prominent YYDxxG motif encoded by a common D gene (IGHD3-22). YYDxxG antibodies demonstrate relatively broad neutralization capabilities, with some antibodies exhibiting avidity-dependent neutralization (20, 28, 29).

The large reservoir of SARS-CoV-2- and SARS-CoV-related sarbecoviruses represents a potential threat for future new zoonotic viral transmissions. Since neutralizing antibodies have been shown during the SARS-CoV-2 pandemic to be very effective in curbing viral disease, as long as they are administered early after symptom onset, a collection of human monoclonal antibodies capable of broadly neutralizing human and bat sarbecoviruses would represent an important first line of defense against future zoonotic outbreaks with one of these viruses.

Here, we describe the isolation of a potent bnAb, pT1679, from a COVID-19 convalescent patient. pT1679 is a YYDxxG antibody capable of neutralizing a broad range of human, bat, and pangolin sarbecoviruses in a pseudotype neutralization assay, and it protects Syrian hamsters against challenge with SARS-CoV-2 Wuhan and Omicron strains. Our results suggest different structural requirements for antibodies that neutralize a broad range of SARS-CoV-2 variants, including SARS-CoV-2 Omicron and those that also target other members of the *Sarbecovirus* genus.

## RESULTS AND DISCUSSION

### Selection of a neutralizing human monoclonal antibody with broad activity against SARS-CoV-2 variants

Previously, we described the screening of sera from SARS-CoV-2-infected patients for neutralizing antibodies in a VSV-pseudotype assay and the subsequent isolation of SARS-CoV-2 spike-reactive B cells from three individuals whose sera neutralized SARS-CoV-2 at high dilutions and also cross-neutralized pseudotypes carrying the S proteins of WIV1, a bat coronavirus, and SARS-CoV (12). To identify antibodies capable of cross-neutralizing other sarbecoviruses, their ability to neutralize SARS-CoV and two bat and two pangolin coronaviruses was tested in the pseudotype neutralization assay.

**Fig 1:**
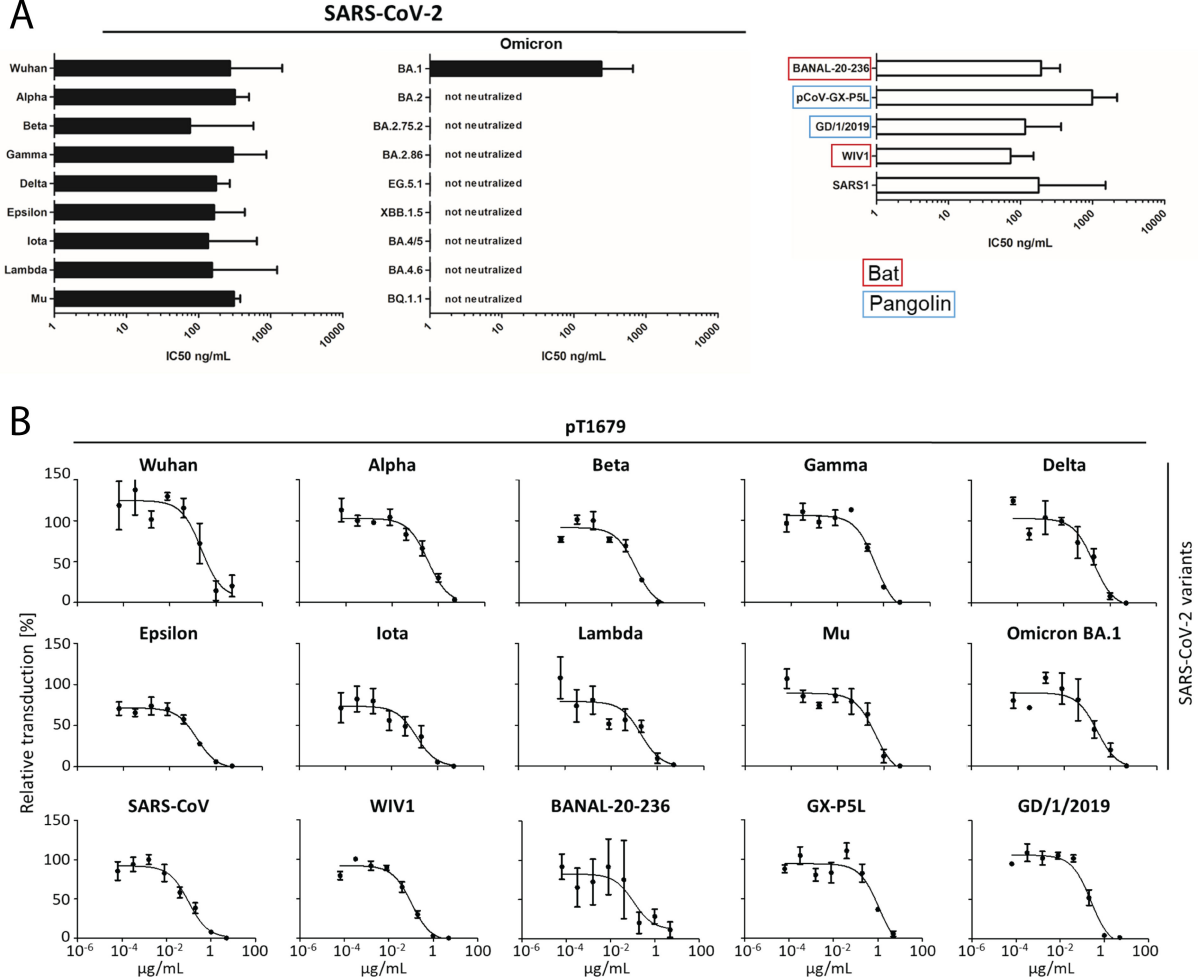
Neutralization of VSV pseudotyped with S proteins from SARS-CoV-2 wild type, SARS-CoV-2 variants, or related sarbecoviruses by pT1679. (**A**) Bar graph showing the geometric mean of the IC50 calculated from three independent experiments, as described in Material and Methods. (**B**) Neutralization graphs for IgG pT1679, showing a representative result of one out of three biological replicate experiments used for the IC50 calculation shown in panel A. While, in general, distinct SARS-CoV-2 neutralization assays produce reproducible neutralization titers, the results can vary due to differences in experimental setup and assay conditions, leading to variations in IC50 values that render it difficult to directly compare these between assays and labs (30).

One cross-reactive antibody (pT1679) from the described B cell sorting (12), featuring a YYDxxG motif in its CDRH3, neutralized all tested SARS-CoV-2-related bat and pangolin sarbecoviruses, as well as SARS-CoV and WIV1, but failed against all tested Omicron variants except for BA.1 (Fig. 1A).

**Fig 2:**
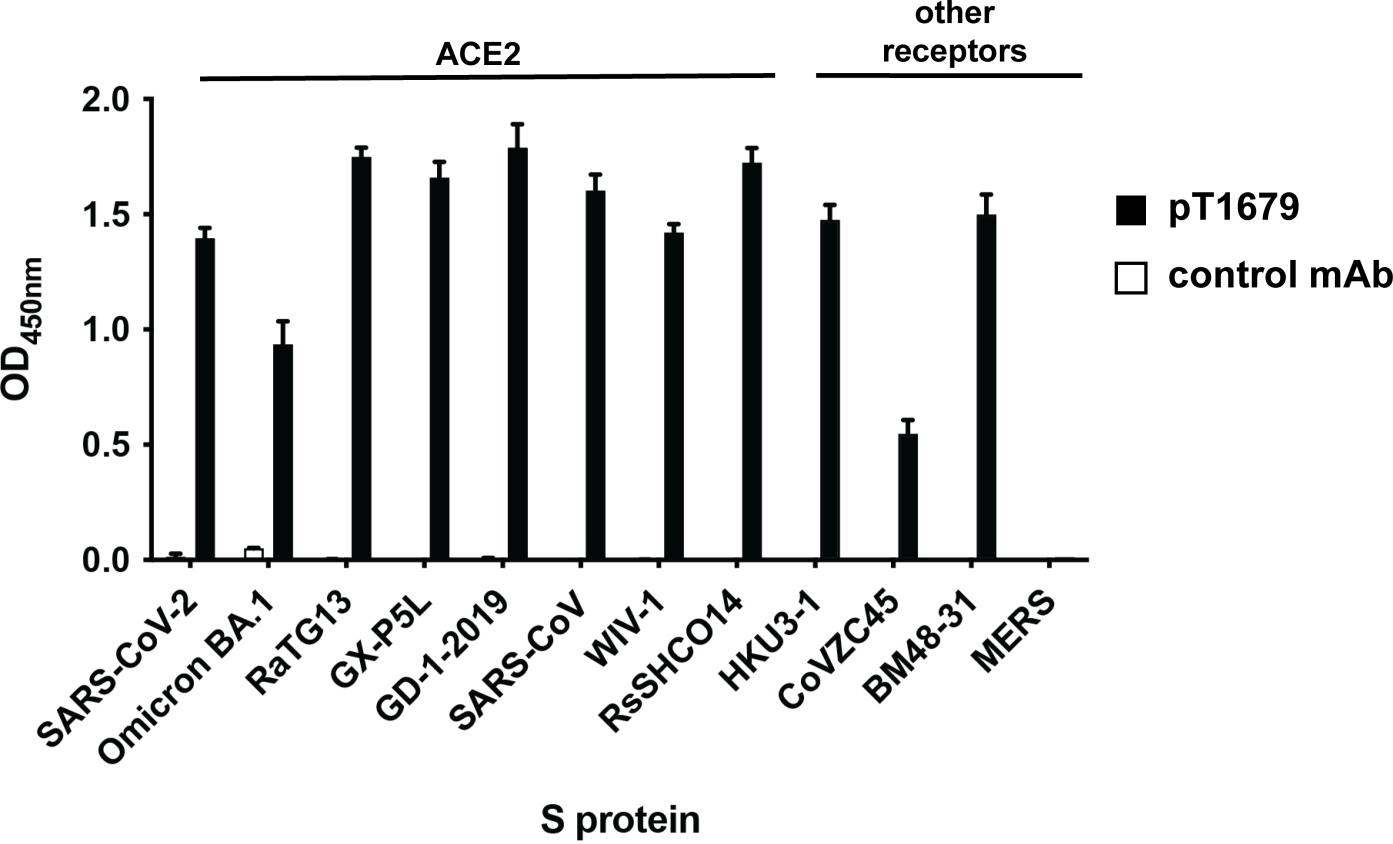
Binding of bnAb pT1679 to selected betacoronavirus S proteins. Binding of pT1679 to recombinant S proteins from 11 representatives of the *Sarbecovirus* subgenus and one from the *Merbecovirus* subgenus was measured by ELISA. S proteins that bind to the angiotensin-converting enzyme 2 (ACE2) or other receptors are indicated. The graph depicts the mean values of four biological replicates (*N* = 4). Error bars indicate the standard deviation.

We further tested the ability of pT1679 to bind to recombinant S proteins from the *Merbecovirus* and *Sarbecovirus* subgenera of the *Betacoronavirus* genus in an ELISA assay (Fig. 2). These also included S proteins from Asian (CoVZC45, HKU3-1, RsSHC014, RaTG13) or European (BM48-31) bat sarbecoviruses, whose S proteins did not readily mediate entry into human cells in our hands and therefore could not be tested in our pseudotype neutralization assay. In keeping with the observed broad neutralization range for related bat and pangolin sarbecoviruses, pT1679 reacted with all tested S proteins apart from that of MERS-CoV. In particular, it also reacted with the S proteins of BM48-31, a European SARS-CoV-related *Sarbecovirus* identified in Bulgarian bats (31), and CoVZC45, a SARS-CoV-related *Sarbecovirus* from Chinese bats (32), which was poorly recognized by previously reported mAbs (33) (Fig. 2). Surface plasmon resonance (SPR) analysis with either trimeric SARS-CoV-2 S ectodomain or recombinant RBD revealed dissociation constants *K_D_* in the picomolar range for the interaction with pT1679 Fab (Table S1). As expected, pT1679 Fab bound with approximately threefold higher affinity to the trimeric S protein compared to binding to the RBD.

### Structural characterization of pT1679

**Fig 3:**
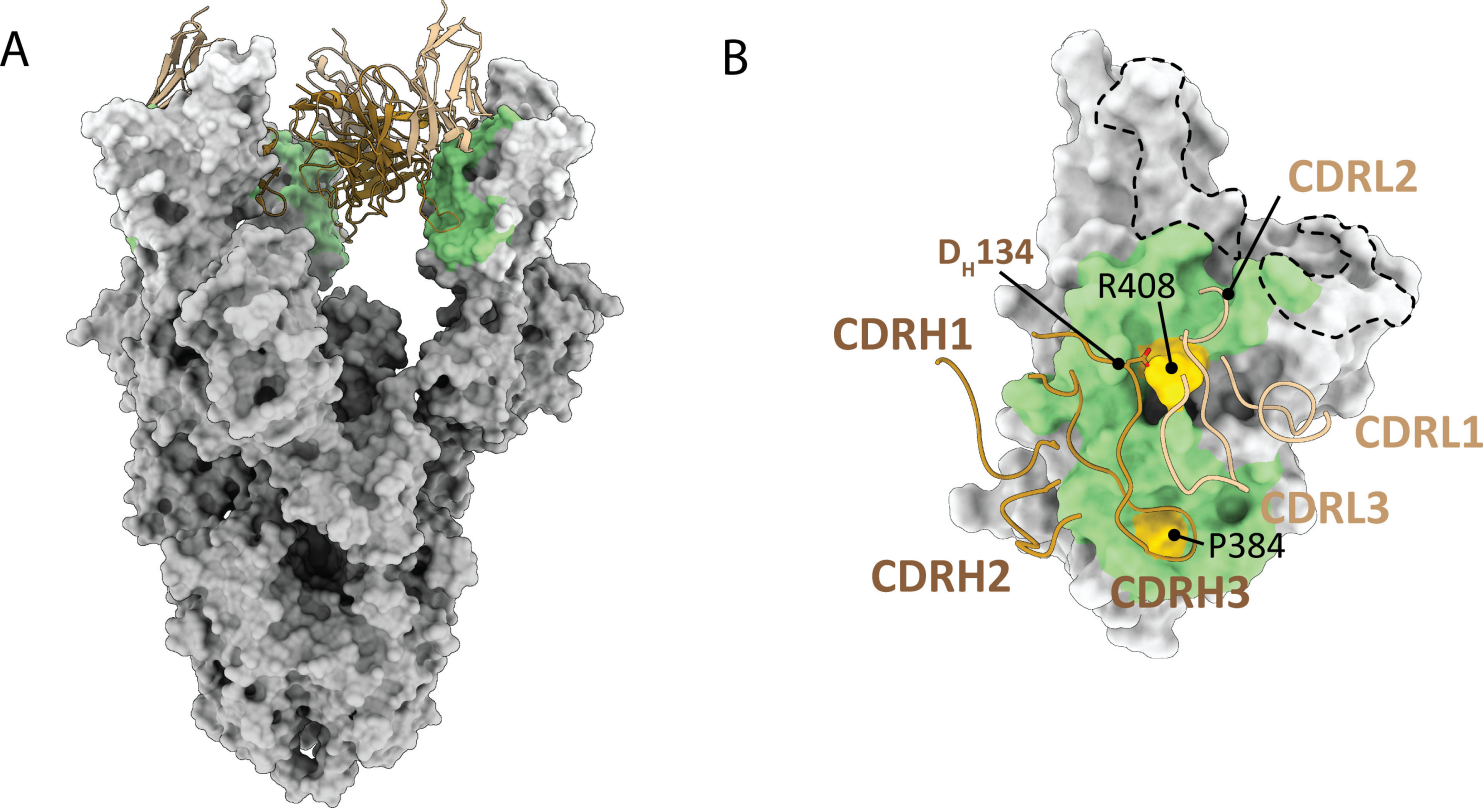
pT1679 targets a conserved extended epitope. (**A**) pT1679 is a class 4 antibody that binds to the S protein in the RBD in “up” conformation. The variable regions of heavy (brown) and light chains (light brown) are shown as cartoon, and the S protein is shown as a white surface with residues in the contact interface highlighted in green. (**B**) The epitope of pT1679 extends towards the ACE2 binding site at the RBD ridge and includes a prominent salt bridge between D_H_134 and R408 (gold). The dashed line indicates RBD interface residues within 2.3 Å of the human ACE2 receptor (6M0J [34]). The long CDRH3 provides a large interaction surface towards the bottom of the RBD contacting P384.

To better understand the interaction of pT1679 with the SARS-CoV-2 S protein, we determined the structure of the corresponding Fab fragment in complex with the trimeric, soluble SARS-CoV-2 S protein ectodomain by cryo-EM to a resolution of 3.27 Å (Table S2, quality of a focused cryo-EM density map shown in Fig. S1). Processing details can be found in Fig. S2. The recognized epitope is located on the inner face of the RBD (Fig. 3) that is buried in the RBD “down” conformation and identifies pT1679 as a Barnes class 4/Cao class F2 antibody (3, 6).

**Fig 4:**
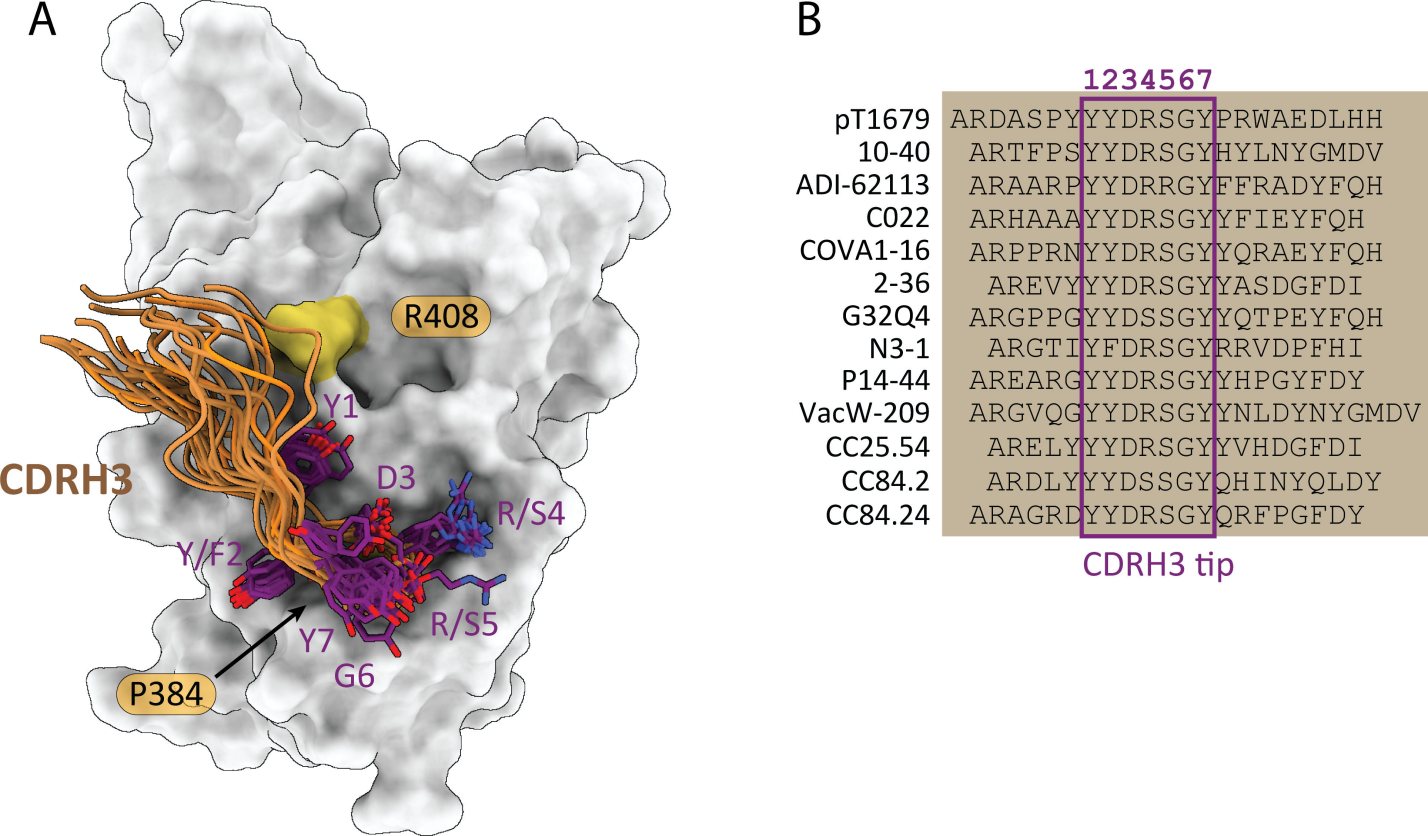
CDRH3 sequence and conformation are shared among several class 4 bnAbs. (**A**) The long CDRH3 of pT1679 and the reported YYDxxG antibodies ADI-62113, C022, COVA1-16, 2-36, N3-1, 10-40, P14-44, VacW-209, G32Q4, CC25.54, CC84.24, and CC84.2 reach towards the bottom of the RBD. The side chains of the seven residues at the tip of CDRH3 are in intimate contact with RBD residue P384 and share a very similar backbone conformation and positioning of side chains. Backbone atoms of CDRH3 are shown as orange cartoon. Side chains of seven residues at the tip of CDRH3 (1–7) are shown in purple (nitrogens: blue; oxygens: red). (**B**) Amino acid alignment of the CDRH3 sequences observed in the previously reported YYDxxG bnAbs .

Although the pT1679 epitope and ACE2 binding site do not overlap extensively, pT1679 is expected to compete with ACE2 binding due to steric hindrance from the positioning of the light chain (Fig. S3). The contact interface is dominated by a long CDRH3 prominently contacting RBD residue P384 with its tip (Fig. 3B). Additional interactions with CDRL2 extend the epitope towards the ACE2 binding site (RBD residues between 403–427 and 505; Fig. 3B; detailed interface characteristics are summarized in Table S3). The interaction with CDRH3 features a salt bridge between D_H_134 and R408, an RBD residue that is mutated to serine in Omicron BA.2, BA.4/5, and other Omicron variants, thus explaining the failure of pT1679 to neutralize these VOCs. In general, the epitope targeted by pT1679 is highly conserved or conservatively substituted between SARS-CoV-2 and other sarbecoviruses (Data S1). Twenty-five antibodies with similar epitopes extending towards the ACE2 binding site have been previously characterized, including 12 YYDxxG antibodies: ADI-62113 (19), C022 (20), COVA1-16 (21), 2-36 (22), N3-1 (23), 10-40 (24), P14-44 (25), VacW-209 (26), G32Q4 (27), CC25.54, CC84.24, and CC84.2 (28). Despite varying V and J gene usage, these antibodies exhibit interaction patterns remarkably similar to pT1679 (Fig. 4A; Table S3). Although N3-1 contains a phenylalanine substitution at the second tyrosine position of the YYDxxG motif (Fig. 4), we included it in our analysis since this conservative amino acid replacement preserves the CDRH3 conformation and maintains similar binding characteristics.

### YYDxxG antibodies fall into two groups with distinct neutralization profiles

**Fig 5:**
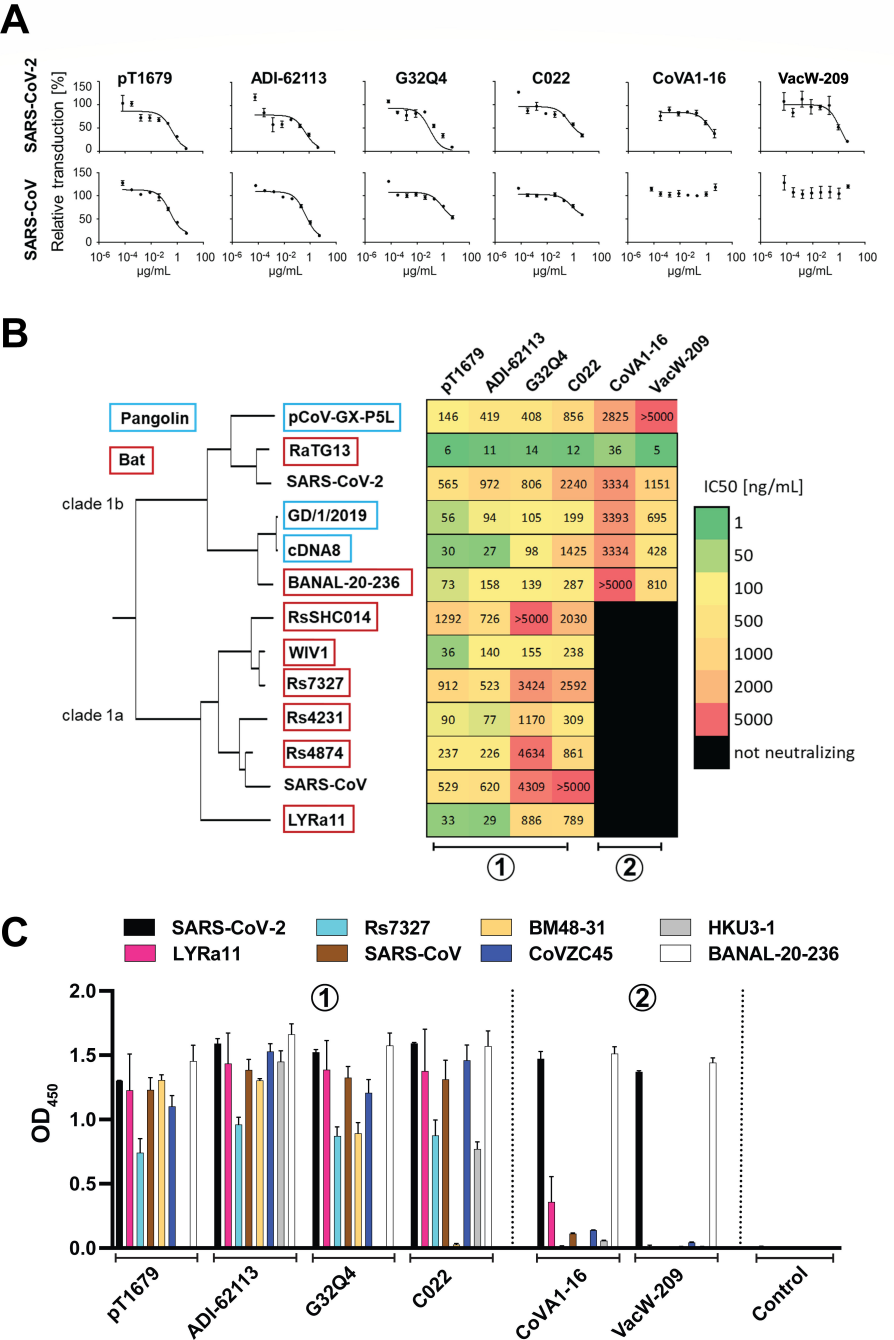
Neutralization and binding profiles of YYDxxG bnAbs. (**A**) Neutralization of VSV pseudotyped with S proteins from SARS-CoV-2 or SARS-CoV on human ACE2 expressing HEK293T cells showing a representative result of one out of three biological replicate experiments used for the IC50 calculation shown in panel B. (**B**) Dendrogram based on the complete protein sequences of S proteins from the shown coronaviruses. Neutralization data shows the geometric mean of the IC50 calculated from three independent experiments. The highest antibody concentration used in these assays was 5 µg/mL. When 50% neutralization was not achieved at this concentration, the result is shown as >5,000 ng/mL. Antibodies that did not achieve any reduction of infectivity at 5 µg/mL are shown as “not neutralizing” (black shading). (C) Binding of YYDxxG antibodies against selected betacoronavirus receptor-binding domains. Binding of YYDxxG group 1 (pT1679, C022, G32Q4, and ADI-62113) and YYDxxG group 2 (CoVA1-16 and VacW-209) antibodies to recombinant S proteins from eight representatives of the *Sarbecovirus* subgenus was measured by ELISA. The graph depicts the mean values of three biological replicates (*N* = 3). Error bars indicate the standard deviation.

Based on the identical binding mode, we expected very similar neutralization profiles for all YYDxxG antibodies. To confirm this, we tested six YYDxxG antibodies (pT1679, ADI-62113, C022, COVA1-16, G32Q4, and VacW-209) for neutralization against a wide range of sarbecoviruses belonging to two of the three previously described *Sarbecovirus* clades (35), including SARS-CoV, SARS-CoV-2, eight bat coronaviruses, and three pangolin coronaviruses, using a VSV pseudotype neutralization assay (Fig. 5A and B) (12). As some of these pseudotype viruses did not infect Vero76 cells sufficiently well to allow neutralization assays, we carried out neutralization assays for all pseudotyped viruses in HEK293T cells overexpressing human ACE2. Unexpectedly, neutralization experiments allowed classification of the tested antibodies into two distinct groups: antibodies in YYDxxG group 1 (pT1679, C022, G32Q4, and ADI-62113) showed a very broad neutralization profile, including all tested pangolin and bat coronaviruses within both clade 1a and clade 1b, whereas neutralization by YYDxxG group 2 antibodies (CoVA1-16 and VacW-209) was limited to clade 1b viruses (Fig. 5A and B). These results were further supported by an ELISA assay with recombinant S proteins from different sarbecoviruses. In line with the neutralization results, YYDxxG group 2 antibodies VacW-209 and CoVA1-16 only recognized S from SARS-CoV-2 and BANAL-20-236, whereas YYDxxG group 1 antibodies showed a much broader profile (Fig. 5C).

### RBD residue P384 is crucial for broad neutralization of YYDxxG antibodies

Given the common binding mode of the YYDxxG antibodies, we next analyzed the amino acid composition of their epitopes within the RBDs of clade 1a and clade 1b sarbecoviruses to understand the different neutralization profiles observed for YYDxxG group 1 and group 2 antibodies. Interestingly, all viruses of clade 1b carry a proline at position 384 (SARS-CoV-2 numbering), whereas clade 1a viruses carry an alanine at this position.

Position 384 has been previously identified as a critical determinant for neutralization of CR3022, an unrelated antibody with a partially overlapping epitope (36). For CR3022, it has been shown that an alanine (as in SARS-CoV) or a proline (as in SARS-CoV-2) at position 384 leads to subtle differences in the positioning of the protein backbone in this region, which, in turn, affects critical hydrogen bonds between RBD and the heavy chain of CR3022 (36). Additional data are available for nAb COVA1-16, with Liu et al. reporting that a P384A replacement in SARS-CoV-2 RBD did not affect COVA-1-16 binding (although a modest affinity loss was detected in biolayer interferometry experiments [29]). On the other hand, Rees-Spear et al. reported complete loss of neutralization for the P384A mutant in an HIV-1 pseudotype neutralization assay (37).

As available structures of both YYDxxG group 1 and group 2 antibody–RBD complexes show that P384 is contacting the tip of the long CDRH3 loop (Fig. 4), it is possible that amino acid replacements at this position have different effects on binding of YYDxxG group 1 and group 2 antibodies. Interestingly, in YYDxxG group 2 antibodies, residue T385 forms a hydrogen bond to the CDRH3 backbone, whereas in YYDxxG group 1 antibodies, no such interaction can be observed (Table S3). It is therefore tempting to speculate that a replacement of proline 384 by alanine as observed in clade 1a viruses induces minor conformational changes of the protein backbone, which, in turn, prevent the formation of a crucial hydrogen bond between the neighboring Thr385 and the CDRH3 of YYDxxG group 2 antibodies. In contrast, YYDxxG group 1 antibodies might not depend on this interaction and thus tolerate amino acid exchanges at this position. However, side-chain positions in available structures are not very well defined due to the lack of high-resolution data, and it is therefore challenging to explain our results on the basis of structural data alone.

**Fig 6:**
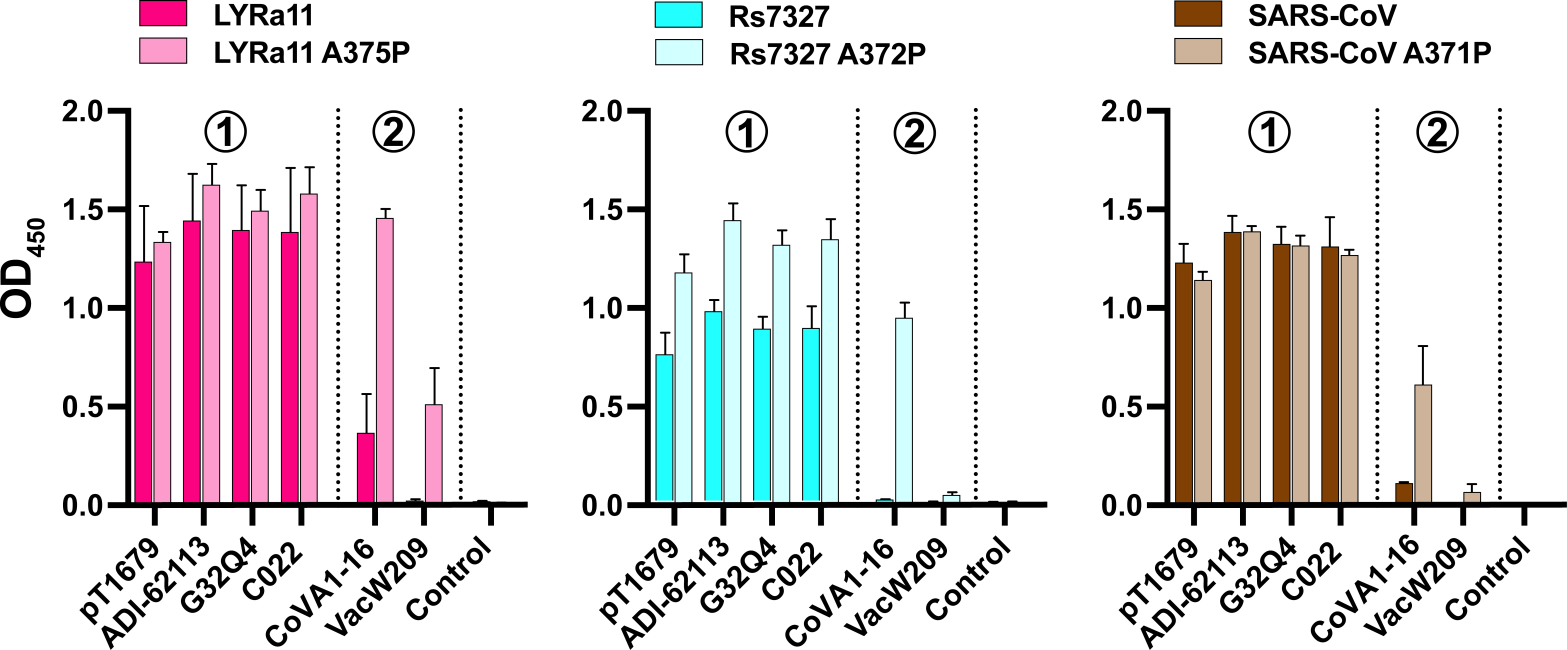
Binding of pT1679-like abs against selected betacoronavirus RBDs. Binding of pT1679-like YYDxxG group 1 and 2 antibodies to recombinant RBDs from LYRa11, Rs7327, and SARS-CoV, as well as corresponding alanine-to-proline mutants at positions equivalent to SARS-CoV-2 residue 384 (LYRa11 A375P, Rs7327 A372P, and SARS-CoV A371P), was measured by ELISA. The graph depicts the mean values of three biological replicates (*N* = 3). Error bars indicate the standard deviation.

To further test the significance of RBD residue 384 (SARS-CoV-2 numbering) for YYDxxG antibody recognition, we introduced alanine-to-proline mutations in the RBDs of SARS-CoV (A371P), LYRa11 (A375P), and Rs7327 (A372P), which are not neutralized by YYDxxG group 2 antibodies CoVA-16 and VacW-209 (Fig. 5A and B). As expected, YYDxxG group 1 antibodies (pT1679, C022, G32Q4, and ADI-62113) showed very similar binding to wild-type RBDs and corresponding mutant RBDs, corroborating that this group tolerates alanine and proline at this position (Fig. 6). Importantly, binding of YYDxxG group 2 antibodies to the tested mutant RBDs, but not the wild-type RBDs, was observed, albeit with marked differences between individual RBDs. While binding of CoVA1-16 to all three tested RBD mutants was comparable to that of YYDxxG group 1 antibodies, VacW-209 showed strong binding to the RBD A375P from LYRa11 (corresponding to P384 in SARS-CoV-2) and increased binding to mutant RBDs from SARS-CoV and Rs7327 only to a much lower extent (Fig. 6). It is possible that additional structural features of the VacW-209 paratope limited its binding to these two RBDs. Our findings indicate that interaction with the amino acid corresponding to position 384 in SARS-CoV-2 RBD is a key factor influencing the neutralization breadth of YYDRxxG antibodies. Further studies are needed to clarify the structural requirements of YYDRxxG group 2 antibodies, such as VacW-209, that target this epitope.

### pT1679 protects against SARS-CoV-2 challenge in a hamster model

**Fig 7:**
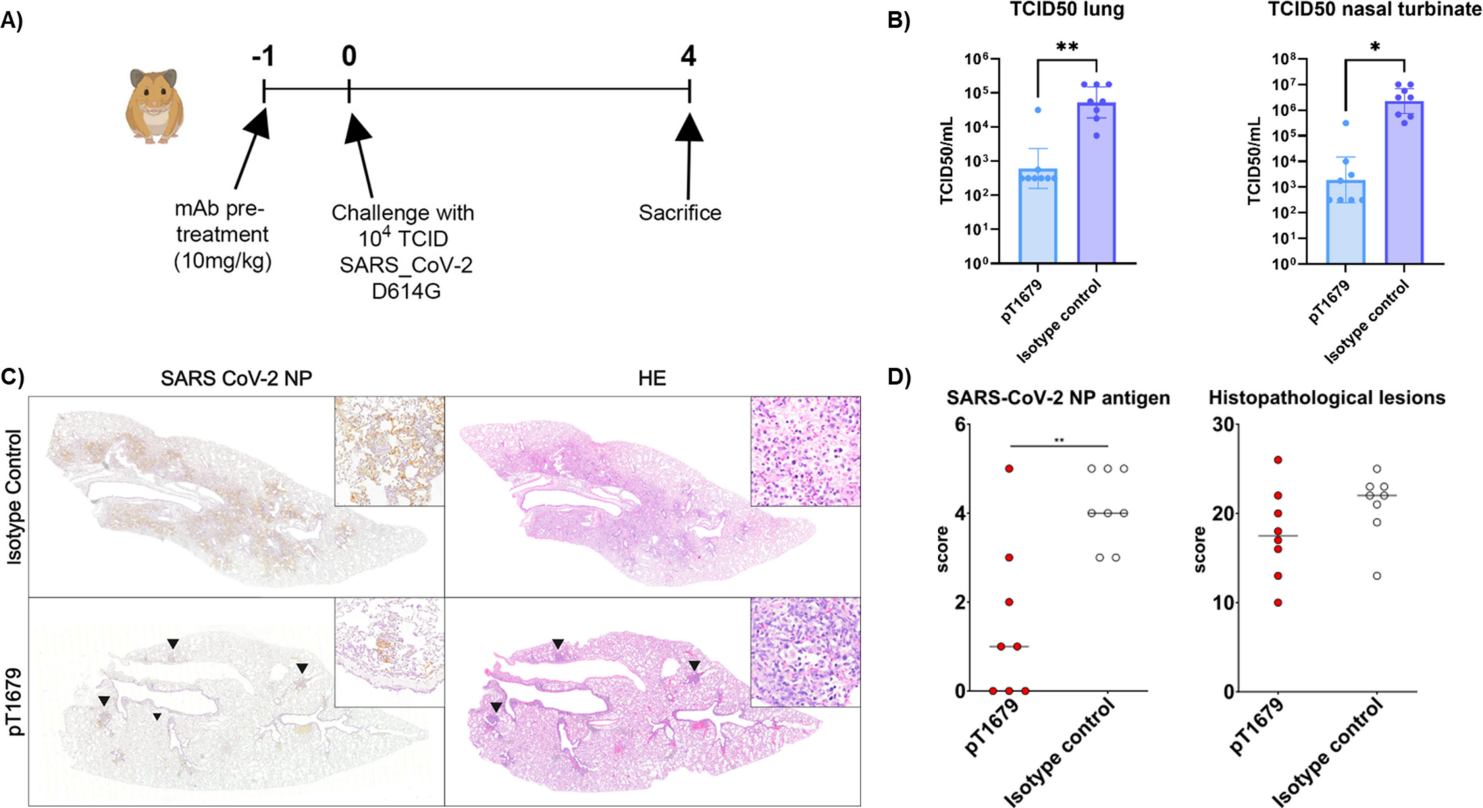
Protection of Syrian hamsters against SARS-CoV-2 infection and disease by nAb pT1679. (**A**) Study design to test the protective effect of pT1679 on SARS-CoV-2 infection. (**B**) Viral load, measured as TCID50, in the lung (left) and nasal turbinates (right) of Syrian hamsters pretreated with 10 mg/kg pT1679 or an isotype control antibody before intranasal challenge with 10^4^ TCID50 of a SARS-CoV-2 B.1/D614G isolate 24 hours later. Data are presented as geometric mean with 95% confidence intervals. Significant differences between control and treated groups are labeled with an asterisk (*P < 0.05, **P < 0.01; unpaired two-tailed *t*-test). (**C**) Immunohistochemistry for SARS-CoV-2 nucleoprotein (NP) (left) and hematoxylin and eosin (H&E) staining (right) of lung tissue sections from animals treated with either the isotype control or the indicated nAbs. Arrowheads indicate SARS-CoV-2 NP immunolabeled cells (left panels) or histopathological lesions characterized by epithelial degeneration and necrosis with immune cell infiltration. (**D**) Semi-quantitative analysis of SARS-CoV-2 immunolabeled cells (left) and histopathological score to assess lesion severity (right) in lung sections of SARS-CoV-2-infected hamsters from the different treatment groups. Significant differences between control and treated groups are labeled with asterisks (*P < 0.05, **P < 0.01, Kruskal–Wallis test).

We next tested whether pT1679 would protect against a SARS-CoV-2 challenge in the hamster model for moderate to severe COVID-19 (38). In a first experiment, we pretreated hamsters by i.p. injection of 10 mg/kg purified pT1679 or an isotype control and challenged them 24 hours later with 10^4^ TCID50 of a SARS-CoV-2 B.1/D614G isolate, as described previously (39) and summarized in Fig. 7A. The animals were sacrificed on day 4. Viral load in lung homogenates and nasal turbinates, measured as TCID50 (40), was reduced by about 2–3 logs in animals pretreated with neutralizing antibody pT1679 compared to isotype control-treated animals (Fig. 7B). To confirm these data and to assess viral antigen distribution within the lung, we immunolabeled lung sections with an antibody against the SARS-CoV-2 nucleoprotein (NP). Isotype control-treated animals showed multifocal areas with numerous immunolabeled pneumocytes (types I and II) and bronchial epithelial cells (Fig. 7C), while animals treated with neutralizing antibodies showed a reduced number of immunolabeled cells in the alveoli (Fig. 7C). Semi-quantitative evaluation of SARS-CoV-2 NP immunolabelings (39) revealed significantly reduced amounts of viral antigen in animals pretreated with antibody pT1679 compared to isotype control-treated animals (Fig. 7D).

On HE-stained lung sections, animals treated with the isotype control antibody showed multifocal, large areas of histopathological changes, including infiltration of immune cells that were affecting alveoli and bronchi (Fig. 7C). These lesions were reduced in animals treated with the SARS-CoV-2-neutralizing antibody pT1679 (Fig. 7C). Lung sections of animals treated with pT1679 showed reduced areas of alveolar histopathological lesions (Fig. 7C). When quantified using a semi-quantitative score (40), these histopathological lesions were reduced in animals pretreated with antibody pT1679, compared to isotype-treated controls (Fig. 7D).

In a second experiment, hamsters were pretreated in a similar manner with antibody pT1679 (at 25 mg/kg) and then challenged with a SARS-CoV-2 Omicron/BA.1 isolate. In this experiment, pT1679 reduced virus replication (TCID50) in the lung by about one log and one and a half logs in the nasal turbinates (Fig. S4A).

Immunolabeling for SARS-CoV-2 NP or histopathology (Fig. S4B) showed that the number of cells expressing SARS-CoV-2 NP and the histopathological scores varied in animals pretreated with the isotype control antibody, with only 3/6 animals showing moderate amounts of viral antigen in the alveoli (Fig. S4C). In the isotype control animals, we found multifocal inflammatory foci affecting bronchi, bronchioles, vessels, and adjacent alveoli (Fig. S4B). Animals treated with bnAbs pT1679 showed only rare immunolabeled cells in the bronchi (arrows and insets in Fig. S4B). In addition, most animals treated with pT1679 showed only focal and minimal inflammatory lesions, restricted to the bronchi (arrows and insets in Fig. S4B).

Together, these results indicate that bnAb pT1679 protects against SARS-CoV-2 replication and SARS-CoV-2-induced histopathological lesions in the respiratory tract of infected hamsters.

### Concluding remarks

In summary, we identified a YYDRxxG antibody (pT1679) capable of binding to a very broad range of *Sarbecovirus* S proteins. This included S proteins of SARS-CoV and its close relative, WIV1, a bat *Sarbecovirus* that infects human cells (41). In addition, pT1679 also neutralized two SARS-CoV-2-related pangolin sarbecoviruses, GD/1/2019 and GX-P5L, as well as BANAL-20-236, a recently reported bat sarbecovirus that is considered to be among the coronaviruses that are most closely related to SARS-CoV-2 (Fig. 1) and can infect human cells (42). The extended CDRH3 of pT1679 interacts with the highly conserved class 4 epitope at the lateral side of the RBD while maintaining crucial interactions with residues in the RBM that have been previously reported to support broad *Sarbecovirus* neutralization.

Our analysis reveals that RBD position 384 (SARS-CoV-2 numbering) serves as a critical determinant that differentiates two classes of YYDRxxG antibodies. YYDxxG group 1 antibodies, including pT1679, accommodate both proline and alanine at position 384, which directly correlates with their broad neutralization capacity. In contrast, YYDxxG group 2 antibodies show strict amino acid specificity at this position, significantly limiting their neutralization breadth. While further structural studies are needed to elucidate the molecular basis for the adaptability of YYDxxG group 1 antibodies, our findings provide a practical framework for predicting the susceptibility of emerging zoonotic sarbecoviruses to YYDRxxG antibodies. We propose that screening candidate antibodies against RBD variants carrying the P384A mutation offers a robust surrogate assay for assessing neutralization breadth. This streamlined approach will be instrumental in accelerating the identification and clinical development of bnAbs capable of targeting diverse sarbecoviruses with pandemic potential, thereby strengthening our preparedness for future outbreaks.

## MATERIALS AND METHODS

### Cell culture

All cell lines were cultivated at 37°C in a humidified atmosphere with 5% CO_2_. HEK293T, HEK293T-ACE2 (cells kindly provided by Markus Hoffmann) (43), Vero76, and BHK-21(G43) were grown in Dulbecco’s modified Eagle medium (DMEM; Gibco 41966-029) with 10% fetal bovine serum (FCS; Sigma F7524). HEK293T-ACE2 cells were supplemented with 0.5 µg/mL puromycin for maintenance of ACE2 expression. Vero76 cells were supplemented with 100 U/mL of penicillin and 0.1 mg/mL of streptomycin (Cytogen, 06-07100). BHK-21(G43) cells were supplemented every fourth passage with 100 µg/mL zeocin and 50 µg/mL hygromycin. Cells were subcultured 1:10 twice ([HEK293T, BHK-21(G43)] or thrice (Vero76) a week. For subculture and seeding of cells, the cells were first washed with phosphate buffered saline (PBS) before detaching them with trypsin/EDTA (Cytogen, 10-023).

### Pseudotype neutralization assays

Expression vectors for the S proteins are listed in Table S5. The codon-optimized DNA coding for coronavirus S proteins lacking the last 18 amino acids to increase infectivity (44) was ordered from GeneArt (Thermo Fisher) and subcloned in the mammalian expression vector pCG1 (Roberto Cattaneo, Mayo Clinic, Rochester, MN, USA) using *Bam*HI and *Xba*I restriction sites. VSV pseudotype neutralization assays were performed, as previously described (45). Briefly, to produce VSV pseudoparticles, 4 × 10^5^ HEK293T cells were transfected via calcium phosphate transfection with 8 µg of the desired surface glycoprotein expression plasmid. Eighteen hours after transfection, the transfected cells were transduced with VSV*ΔG-fLuc(VSV-G), a VSV vector in which the VSV-G open reading frame has been replaced by an expression cassette for eGFP and firefly luciferase and was produced and complemented with VSV-G in BHK-21(G43) cells. An anti-VSV-G antibody (I1, mouse hybridoma supernatant from CRL-2700; ATCC) was used to neutralize VSV-G. Twenty-four hours after transduction, the pseudotype containing supernatant was harvested and used in the neutralization experiments. For these, either 1 × 10^4^ Vero76 cells per well were plated in uncoated 96-well plates, or 4 × 10^4^ HEK293T-ACE2 cells per well were plated in poly-D-lysine-coated 96-well plates the day before the assay. Supernatant containing pseudotype particles was mixed with IgG dilutions and incubated at 37°C for 30 minutes, before being added to the cells in triplicate wells. Twenty hours later, the cells were lysed (1× Lysis-juice, PJK, 102517) for 30 minutes, the lysate was transferred to opaque white plates, and luciferase activity was measured using a firefly luciferase substrate (Beetle-juice, PJK, 102511) and the Orion II (Berthold) or GloMax (Promega) luminometers. The relative transduction efficiency was calculated after deducting background signal from non-infected wells, by setting the signal from pseudotype virus-infected, but not neutralized, wells to 100% and calculating the percentage of the luciferase values for the wells that contained sera or antibodies. To determine IC50 values for individual neutralizing antibodies, we used pseudotype virus preparations with TCID50 values in the range of 10^4^ to 10^5^/mL, with the exception of the BANAL-20-236, RaTG13, and RsSHC014 S protein pseudotype viruses, which were less infective and could only be used at a maximal TCID50 of 10^3^/mL. TCID50 values were determined either on Vero76 cells or HEK293T-ACE2 cells plated in 96-well plates on the day of the neutralization assay. Individual neutralizing IgGs were serially diluted from a starting concentration of 5,000 ng/mL in eight fivefold dilution steps to a concentration of 0.064 ng/mL. Following the subtraction of background firefly luciferase values, IC50 values were calculated using the GraphPad Prism 5’s non-linear regression feature with the log(inhibitor) vs. response (three parameters) equation, with the bottom constrained to zero to account for background subtraction. The heatmap shown in Fig. 5C is based on the geometric mean of the IC50 of three independent experiments.

### Expression and purification of soluble S proteins and SARS-CoV-2 RBD in insect cells

Genes encoding the ectodomains of selected betacoronavirus S proteins (see Table S4) and the respective RBDs (SARS-CoV-2 residues 334–527) were cloned into a pMT vector for expression in *Drosophila* S2 cells, as described previously (46). Expression constructs were designed to allow expression of the SARS-CoV-2, SARS-CoV, and MERS-CoV S trimers in prefusion conformation, as previously described (47–49). However, in addition to a T4 fibritin trimerization motif, a TEV protease cleavage site, a fluorescent protein (mNeon-green), and a double strep-tag were included at the C-terminal end of the ectodomains. All other S proteins were expressed as wild-type proteins. All expression plasmids for the respective RBDs carried an enterokinase (EK) site and a double strep-tag at the C-terminus. All genes were inserted downstream of the BiP signal sequence to allow secretion of the proteins into the S2 cell media. A384P mutations in the respective RBD expression plasmids were generated using QuikChange mutagenesis (Agilent). Stable S2 cell transfectants were established for each construct and the proteins produced, as described previously with minor modifications (50). Briefly, 2 µg S protein expression plasmid was co-transfected with 0.1 µg pCoPuro plasmid (51). Following a six-day selection period, stable cell lines were then adapted to insect-Xpress media (Lonza). For large-scale production, cells were induced with 4 µM CdCl_2_ at a density of 5 × 10^6^ cells/mL for five days, pelleted, and the soluble proteins were affinity-purified from the S2 cell media using a Strep-Tactin XT 4Flow column (IBA Lifesciences). S trimers and all RBD proteins were further purified by size-exclusion chromatography (SEC) using Superose 6 increase and Superdex 75 increase 10/300 columns or Superdex 200 increase 26/600 columns (Cytiva), respectively, equilibrated in 2 mM Tris (pH 8) and 200 mM NaCl (for S proteins) or 20 mM HEPES (pH 7.4) and 150 mM NaCl (for all RBD proteins). Purified proteins were concentrated and stored at −80°C.

### Expression and purification of soluble SARS-CoV-2 S protein trimer in HEK cells

SARS-CoV-2 S HexaPro-coding plasmid was a gift from Jason McLellan (Addgene plasmid #154754). HEK293ExPi cells were transfected using ExpiFectamine 293 transfection reagent (Thermo Fisher Scientific) following the manufacturer’s recommendation with minor modifications. Briefly, a plasmid DNA cocktail containing the SARS-CoV-2 S HexaPro-expression plasmid and plasmids encoding the large T antigen of the SV40 virus as well as the cell cycle inhibitors p21 and p27 (SARS-CoV-2 S HexaPro:p21:p27:SV40 at ratios of 0.69:0.05:0.25:0.01, respectively), was prepared in 5 mL Opti-MEM (Gibco) at a concentration of 1 µg/mL of final culture volume. ExpiFectamine 293 transfection reagent was diluted in 5 mL Opti-MEM and mixed with the plasmid DNA cocktail after five-minute incubation at room temperature. The mixture was then incubated for 20 minutes at room temperature, followed by dropwise addition of the mixtures to the cells. Enhancers were added the next day, and after a four-day expression, period cells were pelleted, and the soluble trimeric S protein was affinity-purified from the supernatant, as described for S proteins from insect cells.

### Expression and purification of Fab

Codon-optimized synthetic genes of Fab sequences, cloned into a pMT vector, were purchased from Twist Biosciences. Constructs carried an EK cleavage site and a double strep-tag at the C-terminus of the Fab heavy chain. Expression of all constructs followed the S2 cell expression protocol described above. The antibody fragments were affinity-purified on Strep-Tactin XT 4Flow column from the S2 cell media followed by SEC using a Superdex 200 increase 10/300 column equilibrated with PBS. Purified proteins were concentrated and stored at −80°C.

### IgG expression and purification

Paired heavy and light chain variable regions of antibodies were amplified from the respective neutralizing Fab and cloned into a pcDNA3.1 expression vector under the control of a CMV promoter. Codon-optimized synthetic genes of the pT1679-like antibodies were purchased from Twist Biosciences and cloned into the same vector. All mAbs were expressed as IgG1 in HEK293ExPi cells as described above, and cells were pelleted five days post-transfection. IgGs were purified by affinity chromatography from the supernatant using a Protein G column (Cytiva), followed by SEC using a Superdex 200 increase 10/300 column equilibrated with PBS. Purified mAbs were concentrated and stored at 4°C.

### ELISA binding assay

Nunc 96 well ELISA plates (Thermo Scientific) were coated with 100 ng of purified S trimers or 125 ng of RBD proteins per well in PBS at 4°C overnight. Plates were washed 3× with 300 µL PBS-T and blocked with 100 µL blocking buffer (PBS-T and 5% skimmed milk) per well for one to two hours at room temperature. The plates were washed once with PBS-T and wells loaded with 50 µL of 2 µg/mL and 0.625 µg/mL antibody diluted in blocking buffer in ELISA experiments with S trimers and RBD proteins, respectively. After a 30–60-minute incubation at room temperature, plates were washed 4× with 300 µL PBS-T, followed by a 30-minute incubation at room temperature with 50 µL per well of horseradish peroxidase-conjugated goat anti-human antibody diluted 1:20,000 to 1:40,000 in blocking buffer. Plates were then washed 4× with PBS-T and developed by adding 100 µL TMB substrate (BioLegend) per well. The reaction was stopped after 10 minutes by addition of 50 µL 1 M H_3_PO_4_, and absorbance was measured at 450 nm with 630 nm as reference using an ELx808 absorbance plate reader (BioTek). Data were analyzed using GraphPad Prism 5.

### Surface plasmon resonance

SPR experiments were performed on a Biacore 3000 (GE Healthcare) at 25°C in running buffer (10 mM HEPES [pH 7.4], 150 mM NaCl, 3 mM EDTA, and 0.05% Tween20). The surface of a CM5 sensor chip (Cytiva) was coated with Strep-TactinXT (IBA Life Sciences) following the amine-coupling protocol from the Twin-Strep-Tag capture kit (IBA Life Sciences). For each analysis cycle (performed in duplicates), the SARS-CoV-2 RBD was injected at a concentration of 3.3 nM for 30 seconds at a flow rate of 10 µL/min, leading to a reproducible immobilization level of 25 RU. Kinetic data were collected by injecting mAbs at concentrations of 75, 50, 25, 10, and 5 nM for 200 seconds at a flow rate of 30 µL/min, followed by washing the chip surface with running buffer until a stable baseline was reached. Regeneration was performed after each cycle by injecting 30 µL of 3 M guanidine hydrochloride at a flow rate of 30 µL/min. To measure kinetics of the mAbs to the S protein, insect cell-expressed SARS-CoV-2 S protein was injected at a concentration of 100 nM for 30 seconds at a flow rate of 10 µL/min, followed by injection of the mAbs, as described above. Data were analyzed using the BiaEvaluation software with fits to the Langmuir binding equation for a 1:1 interaction model.

### Cryo-EM sample preparation, data collection, data processing, and model building

SARS-CoV-2 S HexaPro at 0.34 mg/mL was incubated with five times molar excess of pT1679 at 0.55 mg/mL for five to 10 minutes on ice. 4.5 µL were loaded onto a freshly glow-discharged QF R2/1 Au mesh prior to plunge freezing using a Vitrobot IV (Thermo Fisher Scientific). Data were acquired on a Thermo Fisher Titan Krios transmission electron microscope operated at 300 kV and equipped with a K3 direct electron detector and Gatan BioQuantum energy filter, operated in zero-loss mode with a slit width of 20 eV. Automated data collection was carried out using Thermo Fisher EPU software at a nominal magnification of 130,000× with a pixel size of 0.68 Å in super resolution with binning two and a defocus range of −0.4 to −2.5 µm. The dose rate was adjusted to 15 counts/pixel/second, and each two-second movie was fractionated in 60 frames. After movie frame alignment, estimation of the microscope contrast transfer function (CTF) parameters, and particle picking, particle images were extracted with a box size of 640 × 640 pixel and 4× binned for 2D and initial 3D processing yielding a pixel size of 2.72 Å. Two rounds of reference-free 2D classification were performed to select well-aligned particle stacks for ab initio reconstruction and heterogeneous refinement using the first resulting reconstruction as template in the second round. Selected particle images were subjected to another round of non-uniform refinement along with local CTF refinement and local motion correction. To further improve the density of the Fab variable domains, local refinement was performed using a soft mask encompassing the RBD, NTD, and the Fab variable domains. Next, local resolution estimation, filtering, and sharpening were carried out. Reported resolutions are based on the gold-standard Fourier shell correlation (FSC) of 0.143 criterion. To support model building of pT1679 Fab, maps were post-processed using DeepEMhancer (52). UCSF Chimera (53), Phenix (54), and Coot (55) were used to fit atomic models for the S protein (6XKL or 7LXK) into the cryo-EM maps, and the Fab variable domains were manually built using the structure of an antibody with high sequence similarity (7D × 4) as starting point. Models were refined and relaxed using Phenix and ISOLDE using both sharpened and unsharpened maps (54, 56, 57) and validated using Molprobity (58) and Phenix. See Table S3 for details on data collection, processing, refinement, and validation.

### Structure analysis

Epitopes on the RBD were determined by using PISA (59). UCSF ChimeraX (60) was used to calculate buried surface areas and generate figures.

### Hamster challenge experiment

Approval for animal experiments was given by the German Niedersächsisches Landesamt für Verbraucherschutz und Lebensmittelsicherheit (LAVES file number 20/3493 and 21/375). Syrian hamsters (*Mesocricetus auratus*, 6–10 weeks old, Janvier Labs) were housed under BSL-3 conditions, starting 10 days prior to the experiment. Antibodies were injected intraperitoneally in a volume of 500 µL. The hamsters were challenged intranasally with 10^4^ TCID50 of a SARS-CoV-2 D614G or an Omicron BA.1 isolate. Antibody injection, virus challenge, and euthanasia were performed under isoflurane anesthesia. The animals were monitored for body weight loss and clinical symptoms twice daily until they were humanely euthanized four days after infection.

Infectious SARS-CoV-2 virus particles were quantified, as previously described (39). Left lung lobes from the investigated hamsters were fixed in 10% buffered formalin (Chemie Vertrieb GmbH & Co Hannover KG, Hannover, Germany) and pre-fixed by injections of 10% buffered formalin to ensure an optimal histopathological evaluation (61). Formalin-fixed, paraffin-embedded (FFPE) tissue was used for histology and immunohistochemistry. For the latter, a mouse monoclonal antibody against SARS-CoV-2 NP (Sino Biological, Peking, China; 40143-MM05; dilution 1:16000) was applied overnight at 4°C, followed by the Dako EnVision+ polymer system (Dako Agilent Pathology Solutions) and 3,3′-diaminobenzidine tetrahydrochloride (Sigma-Aldrich, St. Louis, MO, USA), as previously described (40, 62). Hamster lungs were evaluated on one cross-section (at the level of the entry of the main bronchus) and one longitudinal section (along the main bronchus) of the entire left lung lobe. Two semi-quantitative scoring systems, one for the assessment of histopathological lesions (40, 63) and one for viral antigen distribution in the lung (39), were performed, as previously described.

## Data Availability

Cryo-EM maps and coordinates have been deposited in the Protein Data Bank under accession code 9H6U and in the Electron Microscopy Databank under accession code EMD-51901. All other data needed to evaluate the conclusions in this paper are present either in the main text or the supplemental materials.

## AFTER EPUB

*[This article was published on 31 July 2025 with a typographical error in the title. The title was corrected in the current version, posted on 6 August 2025.]*

## SUPPLEMENTAL MATERIAL

Data S1 mbio.00606-25-s0001.xlsx Sarbecovirus RDBs and YYDRxxG antibody epitopes.

Supplemental material mbio.00606-25-s0002.docx Fig. S1 to S4; Tables S1 to S5.

ASM does not own the copyrights to Supplemental Material that may be linked to, or accessed through, an article. The authors have granted ASM a non-exclusive, world-wide license to publish the Supplemental Material files. Please contact the corresponding author directly for reuse.

## References

[1] Tang D, Comish P, Kang R. 2020. The hallmarks of COVID-19 disease. PLoS Pathog 16:e1008536. doi:10.1371/journal.ppat.1008536 32442210 PMC7244094

[2] Vlasova AN, Diaz A, Damtie D, Xiu L, Toh T-H, Lee J-Y, Saif LJ, Gray GC. 2022. Novel canine coronavirus isolated from a hospitalized patient with pneumonia in east Malaysia. Clin Infect Dis 74:446–454. doi:10.1093/cid/ciab456 34013321 PMC8194511

[3] Barnes CO, Jette CA, Abernathy ME, Dam K-MA, Esswein SR, Gristick HB, Malyutin AG, Sharaf NG, Huey-Tubman KE, Lee YE, Robbiani DF, Nussenzweig MC, West AP Jr, Bjorkman PJ. 2020. SARS-CoV-2 neutralizing antibody structures inform therapeutic strategies. Nature 588:682–687. doi:10.1038/s41586-020-2852-1 33045718 PMC8092461

[4] Brouwer PJM, Caniels TG, van der Straten K, Snitselaar JL, Aldon Y, Bangaru S, Torres JL, Okba NMA, Claireaux M, Kerster G, et al.. 2020. Potent neutralizing antibodies from COVID-19 patients define multiple targets of vulnerability. Science 369:643–650. doi:10.1126/science.abc5902 32540902 PMC7299281

[5] Chi X, Yan R, Zhang J, Zhang G, Zhang Y, Hao M, Zhang Z, Fan P, Dong Y, Yang Y, Chen Z, Guo Y, Zhang J, Li Y, Song X, Chen Y, Xia L, Fu L, Hou L, Xu J, Yu C, Li J, Zhou Q, Chen W. 2020. A neutralizing human antibody binds to the N-terminal domain of the Spike protein of SARS-CoV-2. Science 369:650–655. doi:10.1126/science.abc6952 32571838 PMC7319273

[6] Cao Y, Yisimayi A, Jian F, Song W, Xiao T, Wang L, Du S, Wang J, Li Q, Chen X, et al.. 2022. BA.2.12.1, BA.4 and BA.5 escape antibodies elicited by Omicron infection. Nature 608:593–602. doi:10.1038/s41586-022-04980-y 35714668 PMC9385493

[7] Chi X, Xia L, Zhang G, Chi X, Huang B, Zhang Y, Chen Z, Han J, Wu L, Li Z, Sun H, Huang P, Yu C, Chen W, Zhou Q. 2023. Comprehensive structural analysis reveals broad-spectrum neutralizing antibodies against SARS-CoV-2 Omicron variants. Cell Discov 9:37. doi:10.1038/s41421-023-00535-1 37015915 PMC10071473

[8] Huang Q, Han X, Yan J. 2022. Structure-based neutralizing mechanisms for SARS-CoV-2 antibodies. Emerg Microbes Infect 11:2412–2422. doi:10.1080/22221751.2022.2125348 36106670 PMC9553185

[9] He P, Liu B, Gao X, Yan Q, Pei R, Sun J, Chen Q, Hou R, Li Z, Zhang Y, et al.. 2022. SARS-CoV-2 Delta and Omicron variants evade population antibody response by mutations in a single spike epitope. Nat Microbiol 7:1635–1649. doi:10.1038/s41564-022-01235-4 36151403 PMC9519457

[10] Li T, Cai H, Zhao Y, Li Y, Lai Y, Yao H, Liu LD, Sun Z, van Vlissingen MF, Kuiken T, et al.. 2021. Uncovering a conserved vulnerability site in SARS‐CoV‐2 by a human antibody. EMBO Mol Med 13:e14544. doi:10.15252/emmm.202114544 34672091 PMC8646660

[11] Meng L, Zha J, Zhou B, Cao L, Jiang C, Zhu Y, Li T, Lu L, Zhang J, Yang H, Feng J, Gu Z, Tang H, Jiang L, Li D, Lavillette D, Zhang X. 2023. A Spike-destructing human antibody effectively neutralizes Omicron-included SARS-CoV-2 variants with therapeutic efficacy. PLoS Pathog 19:e1011085. doi:10.1371/journal.ppat.1011085 36706160 PMC9907810

[12] Stein SC, Hansen G, Ssebyatika G, Ströh LJ, Ochulor O, Herold E, Schwarzloh B, Mutschall D, Zischke J, Cordes AK, et al.. 2024. A human monoclonal antibody neutralizing SARS-CoV-2 Omicron variants containing the L452R mutation. J Virol 98:e01223-24. doi:10.1128/jvi.01223-24 39494911 PMC11650997

[13] Wang W, Lusvarghi S, Subramanian R, Epsi NJ, Wang R, Goguet E, Fries AC, Echegaray F, Vassell R, Coggins SA, et al.. 2022. Antigenic cartography of well-characterized human sera shows SARS-CoV-2 neutralization differences based on infection and vaccination history. Cell Host Microbe 30:1745–1758. doi:10.1016/j.chom.2022.10.012 36356586 PMC9584854

[14] Yuan M, Wu NC, Zhu X, Lee C-CD, So RTY, Lv H, Mok CKP, Wilson IA. 2020. A highly conserved cryptic epitope in the receptor binding domains of SARS-CoV-2 and SARS-CoV. Science 368:630–633. doi:10.1126/science.abb7269 32245784 PMC7164391

[15] Zhou D, Duyvesteyn HME, Chen C-P, Huang C-G, Chen T-H, Shih S-R, Lin Y-C, Cheng C-Y, Cheng S-H, Huang Y-C, et al.. 2020. Structural basis for the neutralization of SARS-CoV-2 by an antibody from a convalescent patient. Nat Struct Mol Biol 27:950–958. doi:10.1038/s41594-020-0480-y 32737466

[16] Hurt AC, Wheatley AK. 2021. Neutralizing antibody therapeutics for COVID-19. Viruses 13:628. doi:10.3390/v13040628 33916927 PMC8067572

[17] Rosen LE, Tortorici MA, De Marco A, Pinto D, Foreman WB, Taylor AL, Park Y-J, Bohan D, Rietz T, Errico JM, et al.. 2024. A potent pan-sarbecovirus neutralizing antibody resilient to epitope diversification. Cell 187:7196–7213. doi:10.1016/j.cell.2024.09.026 39383863 PMC11645210

[18] Hastie KM, Li H, Bedinger D, Schendel SL, Dennison SM, Li K, Rayaprolu V, Yu X, Mann C, Zandonatti M, et al.. 2021. Defining variant-resistant epitopes targeted by SARS-CoV-2 antibodies: a global consortium study. Science 374:472–478. doi:10.1126/science.abh2315 34554826 PMC9302186

[19] Liu H, Kaku CI, Song G, Yuan M, Andrabi R, Burton DR, Walker LM, Wilson IA. 2022. Human antibodies to SARS-CoV-2 with a recurring YYDRxG motif retain binding and neutralization to variants of concern including Omicron. Commun Biol 5:766. doi:10.1038/s42003-022-03700-6 35906394 PMC9336126

[20] Jette CA, Cohen AA, Gnanapragasam PNP, Muecksch F, Lee YE, Huey-Tubman KE, Schmidt F, Hatziioannou T, Bieniasz PD, Nussenzweig MC, West AP, Keeffe JR, Bjorkman PJ, Barnes CO. 2021. Broad cross-reactivity across sarbecoviruses exhibited by a subset of COVID-19 donor-derived neutralizing antibodies. Cell Rep 36:109760. doi:10.1016/j.celrep.2021.109760 34534459 PMC8423902

[21] Liu H, Yuan M, Huang D, Bangaru S, Zhao F, Lee C-CD, Peng L, Barman S, Zhu X, Nemazee D, Burton DR, van Gils MJ, Sanders RW, Kornau H-C, Reincke SM, Prüss H, Kreye J, Wu NC, Ward AB, Wilson IA. 2021. A combination of cross-neutralizing antibodies synergizes to prevent SARS-CoV-2 and SARS-CoV pseudovirus infection. Cell Host Microbe 29:806–818. doi:10.1016/j.chom.2021.04.005 33894127 PMC8049401

[22] Wang P, Casner RG, Nair MS, Yu J, Guo Y, Wang M, Chan JF-W, Cerutti G, Iketani S, Liu L, Sheng Z, Chen Z, Yuen K-Y, Kwong PD, Huang Y, Shapiro L, Ho DD. 2022. A monoclonal antibody that neutralizes SARS-CoV-2 variants, SARS-CoV, and other sarbecoviruses. Emerg Microbes Infect 11:147–157. doi:10.1080/22221751.2021.2011623 34836485 PMC8725896

[23] Goike J, Hsieh CL, Horton AP, Gardner EC, Zhou L, Bartzoka F, Wang N, Javanmardi K, Herbert A, Abbassi S, et al.. 2023. SARS-COV-2 Omicron variants conformationally escape a rare quaternary antibody binding mode. Commun Biol 6:1250. doi:10.1038/s42003-023-05649-6 38082099 PMC10713552

[24] Liu L, Iketani S, Guo Y, Reddem ER, Casner RG, Nair MS, Yu J, Chan JF-W, Wang M, Cerutti G, et al.. 2022. An antibody class with a common CDRH3 motif broadly neutralizes sarbecoviruses. Sci Transl Med 14:eabn6859. doi:10.1126/scitranslmed.abn6859 35438546 PMC9017343

[25] Zou J, Li L, Zheng P, Liang W, Hu S, Zhou S, Wang Y, Zhao J, Yuan D, Liu L, et al.. 2022. Ultrapotent neutralizing antibodies against SARS-CoV-2 with a high degree of mutation resistance. J Clin Invest 132:e154987. doi:10.1172/JCI154987 35108220 PMC8843702

[26] Ju B, Zheng Q, Guo H, Fan Q, Li T, Song S, Sun H, Shen S, Zhou X, Xue W, Cui L, Zhou B, Li S, Xia N, Zhang Z. 2022. Immune escape by SARS-CoV-2 Omicron variant and structural basis of its effective neutralization by a broad neutralizing human antibody VacW-209. Cell Res 32:491–494. doi:10.1038/s41422-022-00638-6 35260792 PMC8902274

[27] Windsor IW, Tong P, Lavidor O, Moghaddam AS, McKay LGA, Gautam A, Chen Y, MacDonald EA, Yoo DK, Griffths A, Wesemann DR, Harrison SC. 2022. Antibodies induced by an ancestral SARS-CoV-2 strain that cross-neutralize variants from Alpha to Omicron BA.1. Sci Immunol 7:eabo3425. doi:10.1126/sciimmunol.abo3425 35536154 PMC9097876

[28] Yuan M, Wilson IA. 2024. The D gene in CDR H3 determines a public class of human antibodies to SARS-CoV-2. Vaccines (Basel) 12:467. doi:10.3390/vaccines12050467 38793718 PMC11126049

[29] Liu H, Wu NC, Yuan M, Bangaru S, Torres JL, Caniels TG, van Schooten J, Zhu X, Lee C-CD, Brouwer PJM, van Gils MJ, Sanders RW, Ward AB, Wilson IA. 2020. Cross-neutralization of a SARS-CoV-2 antibody to a functionally conserved site is mediated by avidity. Immunity 53:1272–1280. doi:10.1016/j.immuni.2020.10.023 33242394 PMC7687367

[30] Riepler L, Rössler A, Falch A, Volland A, Borena W, von Laer D, Kimpel J. 2020. Comparison of four SARS-CoV-2 neutralization assays. Vaccines (Basel) 9:13. doi:10.3390/vaccines9010013 33379160 PMC7824240

[31] Drexler JF, Gloza-Rausch F, Glende J, Corman VM, Muth D, Goettsche M, Seebens A, Niedrig M, Pfefferle S, Yordanov S, Zhelyazkov L, Hermanns U, Vallo P, Lukashev A, Müller MA, Deng H, Herrler G, Drosten C. 2010. Genomic characterization of severe acute respiratory syndrome-related coronavirus in European bats and classification of coronaviruses based on partial RNA-dependent RNA polymerase gene sequences. J Virol 84:11336–11349. doi:10.1128/JVI.00650-10 20686038 PMC2953168

[32] Hu D, Zhu C, Ai L, He T, Wang Y, Ye F, Yang L, Ding C, Zhu X, Lv R, Zhu J, Hassan B, Feng Y, Tan W, Wang C. 2018. Genomic characterization and infectivity of a novel SARS-like coronavirus in Chinese bats. Emerg Microbes Infect 7:154. doi:10.1038/s41426-018-0155-5 30209269 PMC6135831

[33] Tortorici MA, Czudnochowski N, Starr TN, Marzi R, Walls AC, Zatta F, Bowen JE, Jaconi S, Di Iulio J, Wang Z, et al.. 2021. Broad sarbecovirus neutralization by a human monoclonal antibody. Nature 597:103–108. doi:10.1038/s41586-021-03817-4 34280951 PMC9341430

[34] Lan J, Ge J, Yu J, Shan S, Zhou H, Fan S, Zhang Q, Shi X, Wang Q, Zhang L, Wang X. 2020. Structure of the SARS-CoV-2 spike receptor-binding domain bound to the ACE2 receptor. Nature 581:215–220. doi:10.1038/s41586-020-2180-5 32225176

[35] Starr TN, Zepeda SK, Walls AC, Greaney AJ, Alkhovsky S, Veesler D, Bloom JD. 2022. ACE2 binding is an ancestral and evolvable trait of sarbecoviruses. Nature 603:913–918. doi:10.1038/s41586-022-04464-z 35114688 PMC8967715

[36] Wu NC, Yuan M, Bangaru S, Huang D, Zhu X, Lee C-CD, Turner HL, Peng L, Yang L, Burton DR, Nemazee D, Ward AB, Wilson IA. 2020. A natural mutation between SARS-CoV-2 and SARS-CoV determines neutralization by a cross-reactive antibody. PLoS Pathog 16:e1009089. doi:10.1371/journal.ppat.1009089 33275640 PMC7744049

[37] Rees-Spear C, Muir L, Griffith SA, Heaney J, Aldon Y, Snitselaar JL, Thomas P, Graham C, Seow J, Lee N, Rosa A, Roustan C, Houlihan CF, Sanders RW, Gupta RK, Cherepanov P, Stauss HJ, Nastouli E, Doores KJ, van Gils MJ, McCoy LE, SAFER Investigators. 2021. The effect of spike mutations on SARS-CoV-2 neutralization. Cell Rep 34:108890. doi:10.1016/j.celrep.2021.108890 33713594 PMC7936541

[38] Muñoz-Fontela C, Dowling WE, Funnell SGP, Gsell P-S, Riveros-Balta AX, Albrecht RA, Andersen H, Baric RS, Carroll MW, Cavaleri M, et al.. 2020. Animal models for COVID-19. Nature 586:509–515. doi:10.1038/s41586-020-2787-6 32967005 PMC8136862

[39] Du W, Hurdiss DL, Drabek D, Mykytyn AZ, Kaiser FK, González-Hernández M, Muñoz-Santos D, Lamers MM, van Haperen R, Li W, et al.. 2022. An ACE2-blocking antibody confers broad neutralization and protection against Omicron and other SARS-CoV-2 variants of concern. Sci Immunol 7:eabp9312. doi:10.1126/sciimmunol.abp9312 35471062 PMC9097884

[40] Armando F, Beythien G, Kaiser FK, Allnoch L, Heydemann L, Rosiak M, Becker S, Gonzalez-Hernandez M, Lamers MM, Haagmans BL, Guilfoyle K, van Amerongen G, Ciurkiewicz M, Osterhaus ADME, Baumgärtner W. 2022. SARS-CoV-2 Omicron variant causes mild pathology in the upper and lower respiratory tract of hamsters. Nat Commun 13:3519. doi:10.1038/s41467-022-31200-y 35725735 PMC9207884

[41] Ge X-Y, Li J-L, Yang X-L, Chmura AA, Zhu G, Epstein JH, Mazet JK, Hu B, Zhang W, Peng C, Zhang Y-J, Luo C-M, Tan B, Wang N, Zhu Y, Crameri G, Zhang S-Y, Wang L-F, Daszak P, Shi Z-L, Ge Y. 2013. Isolation and characterization of a bat SARS-like coronavirus that uses the ACE2 receptor. Nature 503:535–538. doi:10.1038/nature12711 24172901 PMC5389864

[42] Temmam S, Vongphayloth K, Baquero E, Munier S, Bonomi M, Regnault B, Douangboubpha B, Karami Y, Chrétien D, Sanamxay D, et al.. 2022. Bat coronaviruses related to SARS-CoV-2 and infectious for human cells. Nature 604:330–336. doi:10.1038/s41586-022-04532-4 35172323

[43] Hoffmann M, Arora P, Groß R, Seidel A, Hörnich BF, Hahn AS, Krüger N, Graichen L, Hofmann-Winkler H, Kempf A, Winkler MS, Schulz S, Jäck H-M, Jahrsdörfer B, Schrezenmeier H, Müller M, Kleger A, Münch J, Pöhlmann S. 2021. SARS-CoV-2 variants B.1.351 and P.1 escape from neutralizing antibodies. Cell 184:2384–2393. doi:10.1016/j.cell.2021.03.036 33794143 PMC7980144

[44] Schwegmann-Weßels C, Glende J, Ren X, Qu X, Deng H, Enjuanes L, Herrler G. 2009. Comparison of vesicular stomatitis virus pseudotyped with the S proteins from a porcine and a human coronavirus. J Gen Virol 90:1724–1729. doi:10.1099/vir.0.009704-0 19264610

[45] Berger Rentsch M, Zimmer G. 2011. A vesicular stomatitis virus replicon-based bioassay for the rapid and sensitive determination of multi-species type I interferon. PLoS One 6:e25858. doi:10.1371/journal.pone.0025858 21998709 PMC3187809

[46] Krey T, d’Alayer J, Kikuti CM, Saulnier A, Damier-Piolle L, Petitpas I, Johansson DX, Tawar RG, Baron B, Robert B, England P, Persson MAA, Martin A, Rey FA. 2010. The disulfide bonds in glycoprotein E2 of hepatitis C virus reveal the tertiary organization of the molecule. PLoS Pathog 6:e1000762. doi:10.1371/journal.ppat.1000762 20174556 PMC2824758

[47] Pallesen J, Wang N, Corbett KS, Wrapp D, Kirchdoerfer RN, Turner HL, Cottrell CA, Becker MM, Wang L, Shi W, Kong WP, Andres EL, Kettenbach AN, Denison MR, Chappell JD, Graham BS, Ward AB, McLellan JS. 2017. Immunogenicity and structures of a rationally designed prefusion MERS-CoV spike antigen. Proc Natl Acad Sci USA 114:E7348–E7357. doi:10.1073/pnas.1707304114 28807998 PMC5584442

[48] Wrapp D, Wang N, Corbett KS, Goldsmith JA, Hsieh CL, Abiona O, Graham BS, McLellan JS. 2020. Cryo-EM Structure of the 2019-nCoV spike in the prefusion conformation. Science. doi:10.1126/science.abb2507 PMC7164637 32075877

[49] Yuan Y, Cao D, Zhang Y, Ma J, Qi J, Wang Q, Lu G, Wu Y, Yan J, Shi Y, Zhang X, Gao GF. 2017. Cryo-EM structures of MERS-CoV and SARS-CoV spike glycoproteins reveal the dynamic receptor binding domains. Nat Commun 8:15092. doi:10.1038/ncomms15092 28393837 PMC5394239

[50] Backovic M, Krey T. 2016. Stable Drosophila cell lines: an alternative approach to exogenous protein expression. Methods Mol Biol 1350:349–358. doi:10.1007/978-1-4939-3043-2_17 26820867

[51] Iwaki T, Figuera M, Ploplis VA, Castellino FJ. 2003. Rapid selection of Drosophila S2 cells with the puromycin resistance gene. BioTechniques 35:482–484. doi:10.2144/03353bm08 14513552

[52] Sanchez-Garcia R, Gomez-Blanco J, Cuervo A, Carazo JM, Sorzano COS, Vargas J. 2021. DeepEMhancer: a deep learning solution for cryo-EM volume post-processing. Commun Biol 4:874. doi:10.1038/s42003-021-02399-1 34267316 PMC8282847

[53] Pettersen EF, Goddard TD, Huang CC, Couch GS, Greenblatt DM, Meng EC, Ferrin TE. 2004. UCSF Chimera--a visualization system for exploratory research and analysis. J Comput Chem 25:1605–1612. doi:10.1002/jcc.20084 15264254

[54] Liebschner D, Afonine PV, Baker ML, Bunkóczi G, Chen VB, Croll TI, Hintze B, Hung LW, Jain S, McCoy AJ, Moriarty NW, Oeffner RD, Poon BK, Prisant MG, Read RJ, Richardson JS, Richardson DC, Sammito MD, Sobolev OV, Stockwell DH, Terwilliger TC, Urzhumtsev AG, Videau LL, Williams CJ, Adams PD. 2019. Macromolecular structure determination using X-rays, neutrons and electrons: recent developments in Phenix. Acta Crystallogr D Struct Biol 75:861–877. doi:10.1107/S2059798319011471 31588918 PMC6778852

[55] Emsley P, Lohkamp B, Scott WG, Cowtan K. 2010. Features and development of Coot. Acta Crystallogr D Biol Crystallogr 66:486–501. doi:10.1107/S0907444910007493 20383002 PMC2852313

[56] Croll TI. 2018. ISOLDE: a physically realistic environment for model building into low-resolution electron-density maps. Acta Crystallogr D Struct Biol 74:519–530. doi:10.1107/S2059798318002425 29872003 PMC6096486

[57] Frenz B, Rämisch S, Borst AJ, Walls AC, Adolf-Bryfogle J, Schief WR, Veesler D, DiMaio F. 2019. Automatically fixing errors in glycoprotein structures with rosetta. Structure 27:134–139. doi:10.1016/j.str.2018.09.006 30344107 PMC6616339

[58] Chen VB, Arendall WB III, Headd JJ, Keedy DA, Immormino RM, Kapral GJ, Murray LW, Richardson JS, Richardson DC. 2010. MolProbity: all-atom structure validation for macromolecular crystallography. Acta Crystallogr D Biol Crystallogr 66:12–21. doi:10.1107/S0907444909042073 20057044 PMC2803126

[59] Krissinel E, Henrick K. 2007. Inference of macromolecular assemblies from crystalline state. J Mol Biol 372:774–797. doi:10.1016/j.jmb.2007.05.022 17681537

[60] Goddard TD, Huang CC, Meng EC, Pettersen EF, Couch GS, Morris JH, Ferrin TE. 2018. UCSF ChimeraX: meeting modern challenges in visualization and analysis. Protein Sci 27:14–25. doi:10.1002/pro.3235 28710774 PMC5734306

[61] Meyerholz DK, Sieren JC, Beck AP, Flaherty HA. 2018. Approaches to evaluate lung inflammation in translational research. Vet Pathol 55:42–52. doi:10.1177/0300985817726117 28812529 PMC5600706

[62] Allnoch L, Beythien G, Leitzen E, Becker K, Kaup F-J, Stanelle-Bertram S, Schaumburg B, Mounogou Kouassi N, Beck S, Zickler M, Herder V, Gabriel G, Baumgärtner W. 2021. Vascular inflammation is associated with loss of aquaporin 1 expression on endothelial cells and increased fluid leakage in SARS-CoV-2 infected golden syrian hamsters. Viruses 13:639. doi:10.3390/v13040639 33918079 PMC8069375

[63] Bošnjak B, Odak I, Barros-Martins J, Sandrock I, Hammerschmidt SI, Permanyer M, Patzer GE, Greorgiev H, Gutierrez Jauregui R, Tscherne A, et al.. 2021. Intranasal delivery of MVA vector vaccine induces effective pulmonary immunity against SARS-CoV-2 in rodents. Front Immunol 12:772240. doi:10.3389/fimmu.2021.772240 34858430 PMC8632543

