# Supplementary material for: A critical residue in a conserved RBD epitope determines neutralization breadth of pan-sarbecovirus antibodies with recurring YYDRxxG motifs
Source: mBio. 2025 Jul 31;16(9):e00606-25. doi: 10.1128/mbio.00606-25 (PMC12421856; doi:10.1128/mbio.00606-25)
Supplement: Supplemental material — Fig. S1 to S4; Tables S1 to S5. [file mbio.00606-25-s0002.docx]

Supplementary Materials for

**A critical residue in a conserved RBD epitope determines neutralization breadth of pan-sarbecovirus antibodies with recurring YYDRxxG motifs**

Saskia C. Stein, George Ssebyatika *et al.*

*Corresponding authors. Email: schulz.thomas@mh-hannover.de; thomas.krey@uni-luebeck.de; guido.hansen@uni-luebeck.de

**This PDF file includes:**

Figs. S1 to S4

Tables S1 to S5

**Supplementary References**


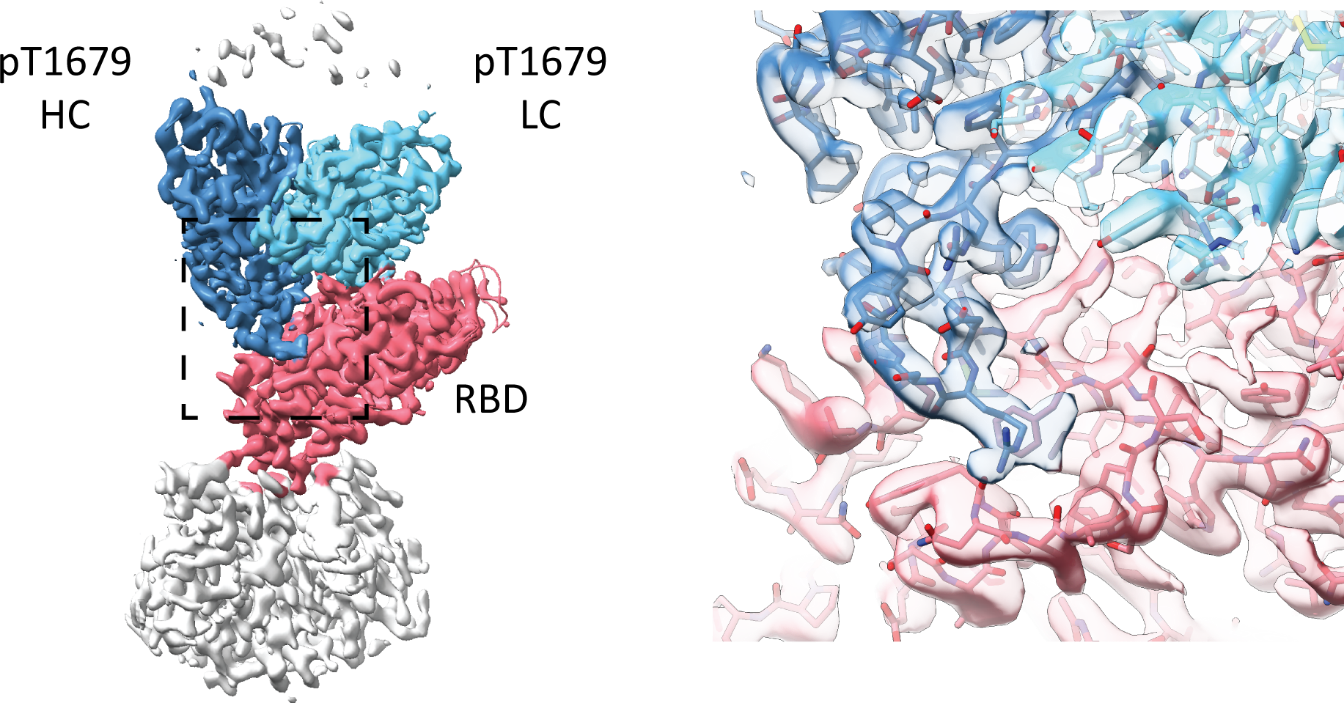


Fig. S1. Cryo-EM density in the PT1679 - S protein complex interface.

Density maps of the epitope-paratope interface of pT1679 with the SARS-CoV-2 RBD (red), the heavy chain is colored in dark blue, the light chain in light blue. The left panel shows overall density of a focused map, the right panel shows details of the antibody-RBD interface with transparent maps and atomic models in stick representation.

**
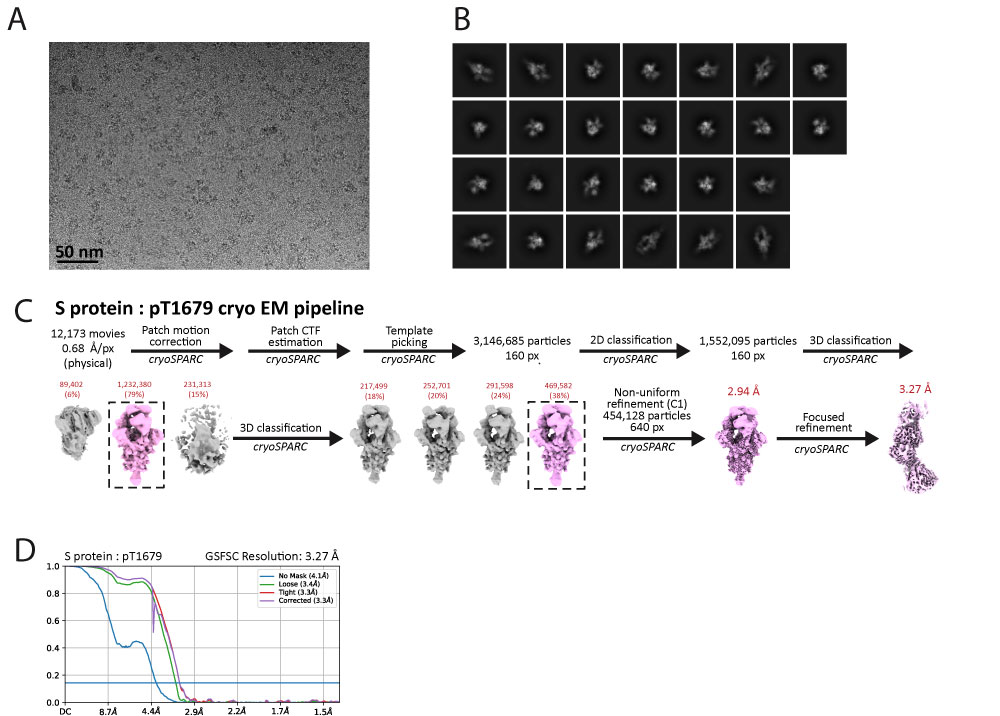
**

Fig. S2. Cryo-EM data processing.

**A)** Representative micrograph showing the distribution of pT1679-SARS-CoV-2 S protein particles in vitreous ice. **B)** Selected two-dimensional class averages. **C)** Simplified flow chart of the cryo-EM processing. One well-resolved class corresponding to a trimeric S protein in the RBD down conformation bound to three pT1679 molecules was identified. Further rounds of classification did not identify additional classes or improve the resolution or map quality. **D)** Fourier shell correlation (FSC) curves.


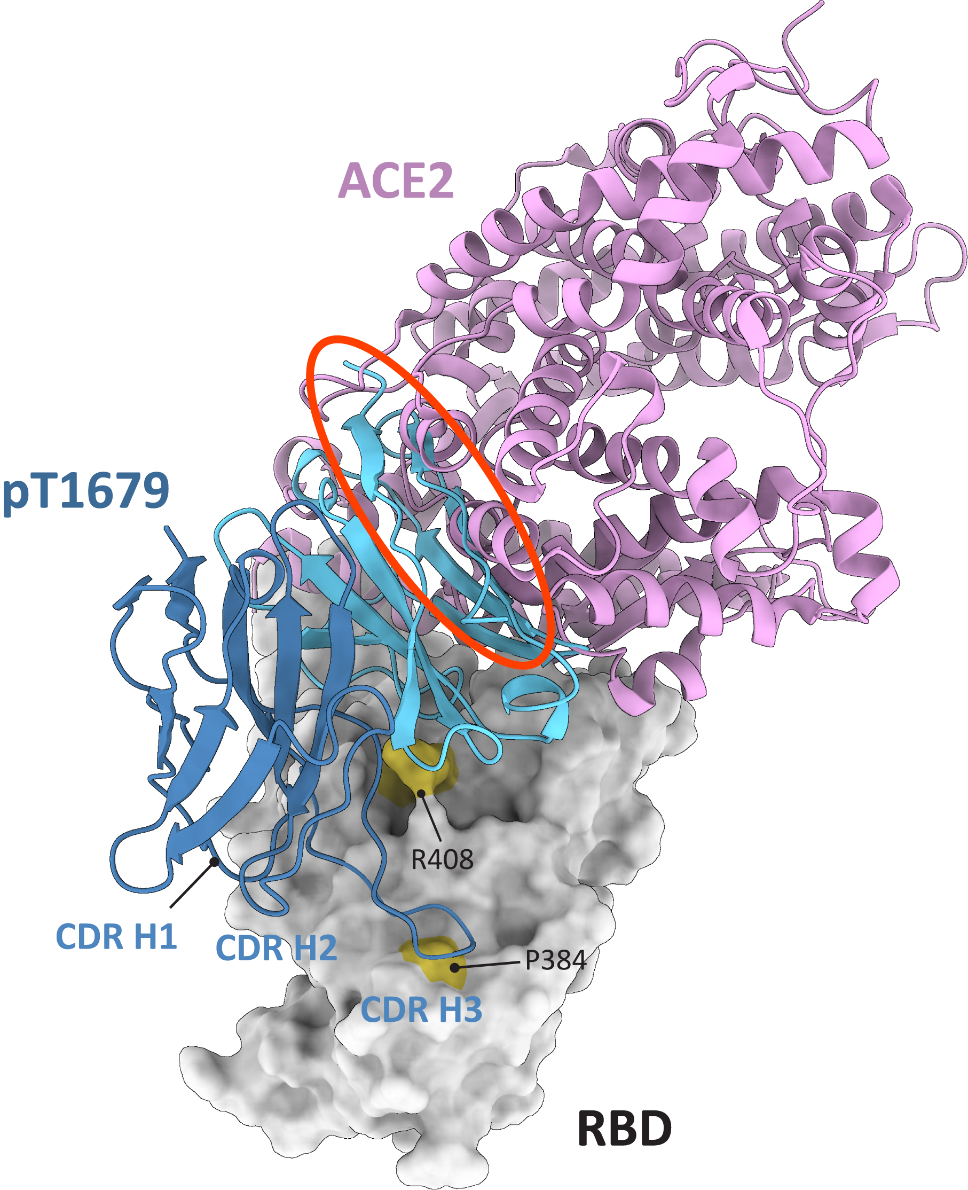


**Figure S3: Overlay of the SARS-CoV-2 RBD complexed to pT1679 and ACE2**

Superposition of pT1679 – S protein complex with the ACE2 - SARS-CoV-2 RBD (6M0J; (1)) complex. The RBD of the pT1679 – S protein complex is shown in surface representation in gray with positions of interface residues P384 and R408 indicated in gold. ACE2 (purple) and pT1679 (HC: dark blue, LC: light blue) are shown in ribbon representation with CDR H1-3 indicated. The region with clashes between the pT1679 LC and the juxtaposed ACE2 is indicated by a red oval.


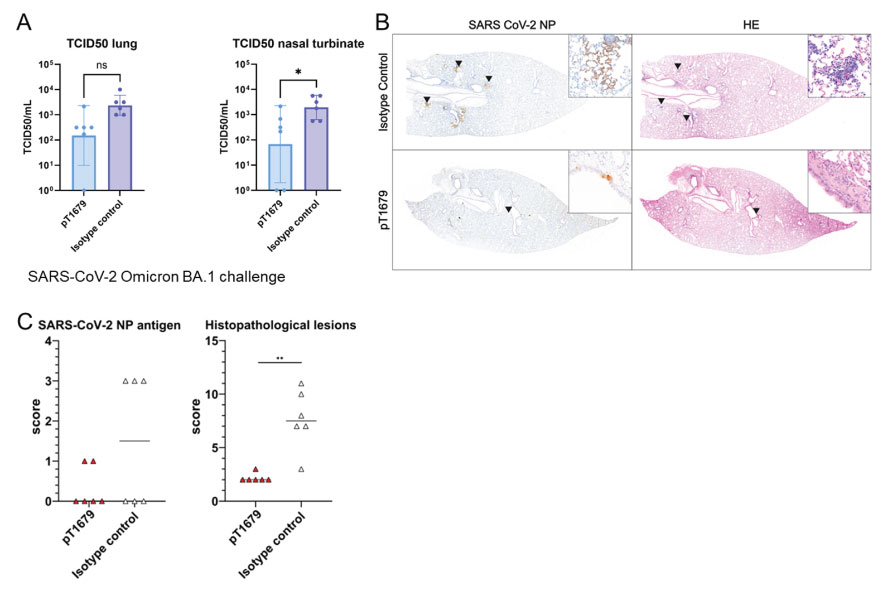


**Figure S4: Protection of Syrian hamsters against SARS-CoV-2 Omicron BA.1 infection and disease by nAb pT1679**.

A) Viral load, measured as TCID50, in the lung (left) and nasal turbinates (right) of Syrian hamsters pretreated with 10 mg/kg pT1679 antibody or an isotype control antibody before intranasal challenge with 10^4^ TCID50 of a SARS-CoV-2 B.1/D614G isolate 24h later. Non detected values were set to 1. Shown is the geometric mean with 95% confidence intervals. Significant differences between control and treated groups are labeled with an asterisk (*P < 0.05; unpaired two-tailed t-test). B) Immunohistochemistry for SARS-CoV-2 nucleoprotein (NP) (left) and Hematoxylin/Eosin (HE) staining (right) of lung tissue sections from animals treated with an isotype control or the indicated nAbs. Arrowheads indicate SARS-CoV-2 NP immunolabelled cells (left panels) or histopathological lesions characterized by epithelial degeneration and necrosis with immune cell infiltration. C) Semi-quantitative analysis of SARS-CoV-2 immunolabelled cells (left), and histopathological score to assess lesion severity (right), in lung sections of SARS-CoV-2 infected hamsters from the different treatment groups. Significant differences between control and treated groups are labeled with asterisks (*p<0.05, ** p<0.01, Kruskal-Wallis test).

|  | **RBD** | | | **S trimer** | | |
| --- | --- | --- | --- | --- | --- | --- |
|  | ***k_a_* (M^-1^s^-1^)** | ***k_d_* (s^-1^)** | ***K_D_* (M)** | ***k_a_* (M^-1^s^-1^)** | ***K_d_* (s^-1^)** | ***K_D_* (M)** |
| **pT1679** | 5 x 10^5^ | 2.6 x 10^-4^ | **5.1 x 10^-10^** | 9 x 10^5^ | 1.2 x 10^-4^ | **1.3 x 10^-10^** |

Table S1. Kinetic parameters of pT1679 binding to the SARS-CoV-2 RBD and trimeric S protein.

| **Complex** | pT1679-S |
| --- | --- |
| PDB | 9H6U |
| EMDB | EMD-51901 |
|  |  |
| **Data collection and processing** |  |
| Number of movies collected | 12,173 |
| Number of frames per movie | 60 |
| Pixel size (Å) | 0.68 |
| Total particle images | 1,552,095 |
|  |  |
| **Refinement** |  |
| Particles in final reconstruction | 454,108 |
| Map resolution (Å), 0.143 FSC | 3.27 |
| Map sharpening B factor (Å²) | -94.7 |
| Number of non-hydrogen atoms | 3,349 |
| Chains | 3 |
| Ligands | 1 |
| Protein | 434 |
| RMS (bonds) | 0.014 |
| RMS (angles) | 1.849 |
|  |  |
| **Validation** |  |
| MolProbity score | 0.95 |
| Ramachandran favored (%) | 96.73 |
| Ramachandran outliers (%) | 0 |
| Rotamer outliers (%) | 0 |
| Clashscore | 0.77 |
|  |  |
| **B factors (Å²)** |  |
| protein | 25.2 |
| ligands | 37.9 |

Table S2. Cryo-EM crystallography data collection, refinement and validation statistics.

|  | **Interface [Å^2^]** | | | | | **HC genes** | | | **LC genes** | |
| --- | --- | --- | --- | --- | --- | --- | --- | --- | --- | --- |
| **nAb** | **Complex** | **H [Å^2^]** | **L [Å^2^]** | **H [%]** | **L [%]** | **V** | **J** | **D** | **V** | **J** |
| **pT1679** | 904 | 567 | 337 | 63 | 37 | IGHV1-69 | IGHJ1 | IGHD3-22 | IGKV1-40 | IGKJ5 |
| **ADI-62113** | 848 | 728 | 155 | 82 | 18 | IGHV1-3 | IGHJ1 | IGHD3-22 | IGKV1-33 | IGKJ2 |
| **C022** | 927 | 743 | 207 | 78 | 22 | IGHV4-39 | IGHJ1 | IGHD3-22 | IGKV1-5 | IGKJ2 |
| **COVA1-16** | 804 | 672 | 145 | 82 | 18 | IGHV1-46 | IGHJ3 | IGHD3-22 | IGKV1-33 | IGKJ4 |
| **Fab2-36** | 864 | 726 | 142 | 84 | 16 | IGHV4-59 | IGHJ6 | IGHD3-22 | IGKV3-20 | IGKJ1 |
| **N3-1** | 843 | 692 | 155 | 82 | 18 | IGHV4-31 | IGHJ3 | IGHD3-22 | IGKV1-5 | IGKJ1 |
| **10-40** | 909 | 706 | 239 | 75 | 25 | IGHV4-39 | IGHJ4 | IGHD3-22 | IGLV6-57 | IGLJ3 |
| **P14-44** | 869 | 713 | 164 | 81 | 19 | IGHV1-46 | IGHJ6 | IGHD3-22 | IGLV2-14 | IGLJ3 |
| **VacW-209** | 971 | 639 | 371 | 63 | 37 | IGHV3-30 | IGHJ1 | IGHD3-22 | IGLV1-40 | IGLJ3 |
| **G32Q4** | 1021 | 670 | 389 | 63 | 37 | IGHV3-30 | IGHJ1 | IGHD3-22 | IGLV1-40 | IGLJ2 |
| **CC25.54** | 880 | 791 | 129 | 86 | 14 | IGHV4-61 | IGHJ6 | IGHD3-22 | IGKV1-5 | IGKJ1 |
| **CC84.24** | 910 | 711 | 215 | 77 | 23 | IGHV3-21 | IGHJ4 | IGHD3-22 | IGLV3-21 | IGLJ3 |
| **CC84.2** | 990 | 599 | 433 | 58 | 42 | IGHV1-3 | IGHJ4 | IGHD3-22 | IGKV3-20 | IGKJ2 |

Table S3. Characteristic features of antibody-RBD interfaces.

^1^ The contribution of individual chains to the antibody-RBD interfaces was analyzed by PISA (2), expressed as percentage of the total interface and calculated using this percentage and the total interface surface.

| **S proteins** | **Origin** | **Genbank accession** |
| --- | --- | --- |
| BM48-31 | bat / Bulgaria | GU190215.1 |
| CoVZC45 | bat / China | MG772933.1 |
| HKU3-1 | bat / China | DQ022305 |
| WIV-1 | bat / China | KC881007 |
| RsSHC014 | bat / China | KC881005 |
| LYRa11 | bat / China | KF569996.1 |
| Rs4231 | bat / China | KY417146.1. |
| Rs4874 | bat / China | KY417150.1 |
| Rs7327 | bat / China | KY417151.1 |
| RaTG13 | bat / Yunnan-China | MN996532 |
| SARS-CoV-2 B1.1.529 | human | UJN96880.1 |
| SARS-CoV | human / China | AY278488 |
| MERS-CoV | human / Saudi Arabia | JX869059 |
| GX-P5L | pangolin / China | MT040333.1 |
| GD/1/2019 | pangolin / China | MT799524.1 |
| cDNA8 | pangolin / Malaysia | QLR06864.1 |
| SARS-CoV-2 | Wuhan-hu-1 | MN908947 |

Table S4. Betacoronaviruses S proteins recombinantly expressed in this study.

| **Plasmid name** | **Pango** | **WHO classification** | **Citation** |
| --- | --- | --- | --- |
| pCAGGS-VSV-G |  |  | (3) |
| pCG1-SARS-2-SΔ18 |  |  | (4) |
| pCG1-SARS-SΔ18 |  |  | (5) |
| pCG1-WIV-1-SΔ18 |  |  | (6) |
| pCG1-MERS-SΔ20 |  |  | (7) |
| pCG1-GD-1-2019-SΔ18 |  |  | (7) |
| pCG1-pCoV-GX-P5L-SΔ18 |  |  | (7) |
| pCG1-BANAL-20-236-SΔ18 |  |  | (7) |
| pCG1_pangolin SL-CoV-SΔ18 (cDNA8) |  |  | (8) |
| pCG1_bat SL-CoV-SΔ18 (LYRa11) |  |  | (8) |
| pCG1_bat SL-CoV-SΔ18 (Rs4231) |  |  | (8) |
| pCG1_bat SL-CoV-SΔ18 (Rs4874) |  |  | (8) |
| pCG1_bat SL-CoV-SΔ18 (Rs7327) |  |  | (8) |
| pCG1-RsSHC014-SΔ18 |  |  | this publication |
| pCG1-SARS-2-SΔ18_mut5 | B.1.1.7 | Alpha | (7) |
| pCG1-SARS-2-SΔ18_mut6 | B.1.351 | Beta | (7) |
| pCG1-SARS-2-SΔ18 (B.1.1.28) | B.1.1.28 | Gamma | (9) |
| pCG1-SARS-2-SΔ18_mut9 | B.1.617.2 | Delta | (7) |
| pCG1-SARS-2-SΔ18_mut7 | B.1.427 | Epsilon | (7) |
| pCG1-SARS-2-SΔ18_mut8 | B.1.526 | Iota | (7) |
| pCG1-SARS-2-SΔ18_mut10 | C.37 | Lambda | (7) |
| pCG1-SARS-2-SΔ18_mut11 | B.1.621 | Mu | (7) |
| pCG1-SARS-2-SΔ18 Omicron BA.1 | B.1.1.529 | Omicron BA.1 | (10) |
| pCG1-SARS-2-SΔ18_mut13 | B.1.1.529.2 | Omicron BA.2 | (7) |
| pCG1-SARS-2-SΔ18 Omicron BA.2.75.2 |  | Omicron BA.2.75.2 | (11) |
| pCDNA3.1_SARS2-Spike BA.4/5 Δ21 | B.1.1.529.4 B.1.1.529.5 | Omicron BA.4/5 | (12) |
| pCG1-SARS-2-SΔ18 Omicron BA.4.6 |  | Omicron BA.4.6 | (13) |
| pCDNA3.1_SARS2-Spike BQ.1.1 Δ21 |  | Omicron BQ.1.1 | (14) |
| pCG1-SARS-2-SΔ18 Omicron XBB.1.5 |  | Omicron XBB.1.5 | (15) |
| pCG1-SARS-2SΔ18 Omicron BA.2.86 |  | Omicron BA.2.86 | (16) |
| pCG1-SARS-2SΔ18 Omicron EG.5.1 |  | Omicron EG.5.1 | (17) |

**Table S5. Plasmids expressing betacoronavirus S proteins used for VSV pseudotype virus generation in this study.**

Data S1. (separate file). Conservation analysis of RBD residues across SARS-CoV-2 VOC/VOI showing contacting residues mAbs with SARS-CoV-2 RBD.

Supplementary references

1. Lan J, Ge J, Yu J, Shan S, Zhou H, Fan S, Zhang Q, Shi X, Wang Q, Zhang L, Wang X. 2020. Structure of the SARS-CoV-2 spike receptor-binding domain bound to the ACE2 receptor. Nature 581:215-220.

2. Krissinel E, Henrick K. 2007. Inference of macromolecular assemblies from crystalline state. J Mol Biol 372:774-97.

3. Brinkmann C, Hoffmann M, Lubke A, Nehlmeier I, Kramer-Kuhl A, Winkler M, Pohlmann S. 2017. The glycoprotein of vesicular stomatitis virus promotes release of virus-like particles from tetherin-positive cells. PLoS One 12:e0189073.

4. Hoffmann M, Kleine-Weber H, Pohlmann S. 2020. A Multibasic Cleavage Site in the Spike Protein of SARS-CoV-2 Is Essential for Infection of Human Lung Cells. Mol Cell 78:779-784 e5.

5. Hoffmann M, Muller MA, Drexler JF, Glende J, Erdt M, Gutzkow T, Losemann C, Binger T, Deng H, Schwegmann-Wessels C, Esser KH, Drosten C, Herrler G. 2013. Differential sensitivity of bat cells to infection by enveloped RNA viruses: coronaviruses, paramyxoviruses, filoviruses, and influenza viruses. PLoS One 8:e72942.

6. Vanshylla K, Fan C, Wunsch M, Poopalasingam N, Meijers M, Kreer C, Kleipass F, Ruchnewitz D, Ercanoglu MS, Gruell H, Munn F, Pohl K, Janicki H, Nolden T, Bartl S, Stein SC, Augustin M, Dewald F, Gieselmann L, Schommers P, Schulz TF, Sander LE, Koch M, Luksza M, Lassig M, Bjorkman PJ, Klein F. 2022. Discovery of ultrapotent broadly neutralizing antibodies from SARS-CoV-2 elite neutralizers. Cell Host Microbe 30:69-82 e10.

7. Stein SC, Hansen G, Ssebyatika G, Stroh LJ, Ochulor O, Herold E, Schwarzloh B, Mutschall D, Zischke J, Cordes AK, Schneider T, Hinrichs I, Blasczyk R, Kleine-Weber H, Hoffmann M, Klein F, Kaiser FK, Gonzalez-Hernandez M, Armando F, Ciurkiewicz M, Beythien G, Pohlmann S, Baumgartner W, Osterhaus A, Schulz TF, Krey T. 2024. A human monoclonal antibody neutralizing SARS-CoV-2 Omicron variants containing the L452R mutation. J Virol 98:e0122324.

8. Zhang L, Cheng HH, Kruger N, Hornich B, Graichen L, Hahn AS, Schulz SR, Jack HM, Stankov MV, Behrens GMN, Muller MA, Drosten C, Morer O, Winkler MS, Qian Z, Pohlmann S, Hoffmann M. 2024. ACE2-independent sarbecovirus cell entry can be supported by TMPRSS2-related enzymes and can reduce sensitivity to antibody-mediated neutralization. PLoS Pathog 20:e1012653.

9. Hoffmann M, Arora P, Gross R, Seidel A, Hornich BF, Hahn AS, Kruger N, Graichen L, Hofmann-Winkler H, Kempf A, Winkler MS, Schulz S, Jack HM, Jahrsdorfer B, Schrezenmeier H, Muller M, Kleger A, Munch J, Pohlmann S. 2021. SARS-CoV-2 variants B.1.351 and P.1 escape from neutralizing antibodies. Cell 184:2384-2393 e12.

10. Arora P, Zhang L, Krüger N, Rocha C, Sidarovich A, Schulz S, Kempf A, Graichen L, Moldenhauer A-S, Cossmann A, Dopfer-Jablonka A, Behrens GMN, Jäck H-M, Pöhlmann S, Hoffmann M. 2022. SARS-CoV-2 Omicron sublineages show comparable cell entry but differential neutralization by therapeutic antibodies. Cell Host & Microbe 30:1103-1111.e6.

11. Arora P, Kempf A, Nehlmeier I, Schulz SR, Jack HM, Pohlmann S, Hoffmann M. 2023. Omicron sublineage BQ.1.1 resistance to monoclonal antibodies. Lancet Infect Dis 23:22-23.

12. Gruell H, Vanshylla K, Korenkov M, Tober-Lau P, Zehner M, Münn F, Janicki H, Augustin M, Schommers P, Sander LE, Kurth F, Kreer C, Klein F. 2022. SARS-CoV-2 Omicron sublineages exhibit distinct antibody escape patterns. Cell Host & Microbe 30:1231-1241.e6.

13. Arora P, Zhang L, Nehlmeier I, Kempf A, Cossmann A, Dopfer-Jablonka A, Schulz SR, Jack HM, Behrens GMN, Pohlmann S, Hoffmann M. 2022. The effect of cilgavimab and neutralisation by vaccine-induced antibodies in emerging SARS-CoV-2 BA.4 and BA.5 sublineages. Lancet Infect Dis 22:1665-1666.

14. Dewald F, Pirkl M, Paluschinski M, Kuhn J, Elsner C, Schulte B, Knufer J, Ahmadov E, Schlotz M, Oral G, Bernhard M, Michael M, Luxenburger M, Andree M, Hennies MT, Hafezi W, Muller MM, Kumpers P, Risse J, Kill C, Manegold RK, von Frantzki U, Richter E, Emmert D, Monzon-Posadas WO, Graff I, Kogej M, Buning A, Baum M, Teipel F, Mochtarzadeh B, Wolff M, Gruell H, Di Cristanziano V, Burst V, Streeck H, Dittmer U, Ludwig S, Timm J, Klein F. 2023. Impaired humoral immunity to BQ.1.1 in convalescent and vaccinated patients. Nat Commun 14:2835.

15. Hoffmann M, Arora P, Nehlmeier I, Kempf A, Cossmann A, Schulz SR, Morillas Ramos G, Manthey LA, Jack HM, Behrens GMN, Pohlmann S. 2023. Profound neutralization evasion and augmented host cell entry are hallmarks of the fast-spreading SARS-CoV-2 lineage XBB.1.5. Cell Mol Immunol 20:419-422.

16. Zhang L, Kempf A, Nehlmeier I, Cossmann A, Richter A, Bdeir N, Graichen L, Moldenhauer AS, Dopfer-Jablonka A, Stankov MV, Simon-Loriere E, Schulz SR, Jack HM, Cicin-Sain L, Behrens GMN, Drosten C, Hoffmann M, Pohlmann S. 2024. SARS-CoV-2 BA.2.86 enters lung cells and evades neutralizing antibodies with high efficiency. Cell 187:596-608 e17.

17. Zhang L, Kempf A, Nehlmeier I, Cossmann A, Dopfer-Jablonka A, Stankov MV, Schulz SR, Jack HM, Behrens GMN, Pohlmann S, Hoffmann M. 2023. Neutralisation sensitivity of SARS-CoV-2 lineages EG.5.1 and XBB.2.3. Lancet Infect Dis 23:e391-e392.
